# Spatiotemporally Controlled Sequential Chemo‐Immunotherapy Activates Pyroptosis for Robust Anti‐Tumor Immunotherapy

**DOI:** 10.1002/advs.77722

**Published:** 2026-09-16

**Authors:** Suhui Sun, Ruiqi Wu, Qingshuang Tang, Yunli Xu, Wanrui Shi, Mengxin Wang, Haonan Wang, Yan Luo, Cheng Ma, Xiaolong Liang

**Affiliations:** ^1^ Beijing Key Laboratory of Ultrasound Targeted Drug Delivery and Frontier Imaging Technology, Department of Ultrasound Peking University Third Hospital Beijing People's Republic of China; ^2^ State Key Laboratory of Vascular Homeostasis and Remodeling, Beijing Key Laboratory of Interdisciplinary Research in Gastrointestinal Oncology (BLGO) Peking University Third Hospital Beijing People's Republic of China; ^3^ General Ultrasound Department Beijing Anzhen Hospital Capital Medical University Beijing People's Republic of China; ^4^ Department of Ultrasound The First Affiliated Hospital of Nanchang University Nanchang People's Republic of China; ^5^ Department of Electronic Engineering Tsinghua University Beijing People's Republic of China

**Keywords:** chemotherapy, sequential drug release, synergistic anti‐tumor immunity, ultrasound

## Abstract

Cancer immunotherapy can be augmented by combination of immune checkpoint inhibitors (ICIs) and chemotherapy. However, the administration pharmacokinetics, timing and sequencing for chemotherapy and immunotherapy affect the synergistic outcomes of a combined therapy. Currently, the stable co‐delivery and long ‐termretention of chemotherapy and immunotherapy drugs into the tumor while well control their respective release at an appropriate time intervals is still challenging. Here, we report a nanotherapeutic strategy to augment tumor immunotherapy using highly stable cerasome nanoparticles that are co‐loaded with the ICIs agent, BMS1166, and the chemotherapeutic drug, gemcitabine. Gemcitabine is released under high level of glutathione (GSH) conditions, leading to effective immunogenic cell death, pyroptosis and high PD‐L1 expression on the tumor. Then the local ultrasound irradiation at an optimal time triggers the release of BMS1166 to blockade the PD‐L1, eliciting a potent and durable anti‐tumor immune response. This dual response strategy enables sequentially controlled drug release in the tumor to ensure the same spatiotemporal distribution of different drugs, while playing their respective roles at different times, greatly enhancing the immunotherapy efficiency. In tumor‐bearing‐mice, treatment with the nanoparticles effectively suppresses primary tumors and distant tumors, while preventing tumor metastasis and causing immune memory effect.

## Graphical abstract

A glutathione and ultrasound dual‐responsive cerasome achieves sequential co‐delivery of gemcitabine and BMS1166 in tumors. Chemotherapy priming followed by on‐demand PD‐L1 blockade synergistically amplifies anti‐tumor immunity, inhibiting primary tumor growth, distant metastasis and inducing durable immune memory.

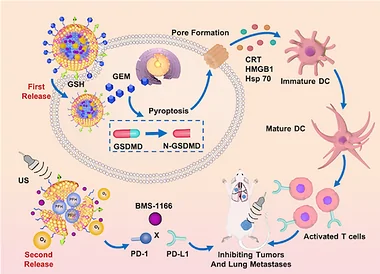

## 1 Introduction

Cancer immunotherapy utilizing the immune system to combat malignant tumors has repeatedly demonstrated efficacy, which attracts extensive attention and has recently been exploited in clinic [1, 2]. Especially, immune checkpoint blockade (ICB) therapy action by hindering the contact of programmed‐cell‐death protein 1 (PD‐1) on cytotoxic T lymphocytes (T cells) with programmed‐death ligand 1 (PD‐L1 and PD‐L2) on tumor cells has achieved great success, exhibiting durable tumor regression and long‐term remissions in various tumors with established immune evasion and/or tolerance [3, 4, 5, 6]. Despite having unique advantages, only a subset of patients (only about 20%–30%) show good clinical responses to this therapy [7, 8, 9, 10, 11], which is due to the low immunogenicity of tumor with characteristics of immunosuppressive tumor microenvironment [12, 13, 14, 15, 16] and insufficient tumor infiltration of cytotoxic T cells [17, 18]. Therefore, efforts to improve the ICB response rate for tumor is still the major challenge in cancer immunotherapy.

To magnify ICB therapeutic efficacy, immune checkpoint inhibitors (ICIs) combined with chemotherapy has been widely applied in clinic, revealing augmented tumor immunogenicity and enhanced ICB response rate [19, 20, 21, 22, 23]. In addition to wide application in clinic by directly attack cancer cells, chemotherapy also possesses different immunomodulatory effects in enhancing tumor killing, including induction of tumor immunogenic cell death (ICD) [24, 25, 26], activation of tumor antigen‐specific CD8+ T cells [27, 28], and regulation of PD‐L1/PD‐1 expressions on cancer/T cells by the danger signaling molecule, such as high‐mobility group protein B1 [29]. Despite promising results, current clinical chemotherapy drugs are mainly the liposomal formulations (such as FDA approved Abraxane, Marqibo, Doxil, and DaunoXome) [30, 31, 32], exhibiting certain reduced side effects and improved tumor accumulation of chemotherapeutic agents, achieving better tumor chemotherapy [33, 34, 35]. However, liposomal drugs often exhibit insufficient morphological stability in blood circulation, where plasma proteins and blood flow shear stress may cause disruption of liposomes, leading to premature drug leakage, thus greatly reduce therapeutic efficiency while cause damage to healthy organs [36, 37, 38, 39]. Similarly, ICB therapy using immune checkpoint inhibitors (ICIs) of anti‐PD‐L1 antibodies (aPD‐L1) usually suffer from off‐target binding to the normal tissues when systemically administrated, since PD‐L1 is also expressed on vascular endothelium, epithelium, hepatocytes, pancreatic islet cells, mesenchymal stem cells, and muscle [40, 41], which not only compromised the therapeutic efficacy but also leading to severe immune‐related adverse events [42, 43]. In addition, chemotherapy and immunotherapy drugs in different forms are often administered separately, resulting in different in vivo temporal and spatial distributions of the drugs [44, 45, 46], and the timing and pharmacological peaking action for the different drugs varies greatly [47, 48], making it difficult to achieve consistent action sites and timely and accurate synergistic therapeutic effect.

More importantly, different chemotherapy and immunotherapy combination strategies may result in different antitumor effects [49, 50]. Immunotherapy followed by chemotherapy may result in the low immune response rate as mentioned above in “immune‐cold” tumors [19, 20, 21, 22, 23]; Simultaneously administering immunotherapy and chemotherapy may increase the immune response rate, but chemotherapy drugs may simultaneously kill activated T lymphocytes, leading to a decrease in the effectiveness of immunotherapy [47, 48]; Chemotherapy followed by immunotherapy can first transform “immune‐cold” tumors into “immune‐hot” tumors, improving the immune response rate to ICB treatment, but the timing of immunotherapy administration is particularly critical [49, 50]. Thus, the optimal combination timing of these two treatment modalities plays leading role in improving the tumor therapeutic outcomes. However, randomized arrangement or followed by established regimen for the administration timing and sequencing of chemotherapy and immunotherapy are performed in current clinical practice, causing much uncertainty and leaving optimization space. Therefore, achieving stable co‐delivery of chemotherapy and immunotherapy drugs and long‐term retention in tumors using stable and biocompatible liposomal nanocarriers, while sequentially controlling the release of different drugs at optimized time intervals as needed, are crucial for improving immune efficacy.

A critical requirement for sequential release is that the carrier must retain ICIs and chemotherapy drug during systemic circulation while sequentially control their release upon reaching the tumor site. Conventional liposomes, despite their widespread use in drug delivery, suffer from inherent instability that can lead to premature drug leakage. In contrast, the unique organosilica hybrid shell of cerasomes provides superior structural stability and drug retention, which are essential for maintaining carrier integrity during circulation and enabling sequential release [51, 52, 53, 54].

We also compared the existing ultrasound‐responsive nano‐immuno‐chemotherapy systems. Current cerasome formulations are mainly used for single‐drug delivery; extending cerasomes to systems capable of simultaneously delivering chemotherapeutic agents and immune checkpoint inhibitors represents a significant advance. Most existing systems rely solely on ultrasound stimulation for drug release without utilizing endogenous glutathione [55, 56, 57]. Although several GSH/ultrasound dual‐responsive nanosystems have been developed, these carriers are typically conventional liposomes, polymers, or exosomes, lacking the structural stability and anti‐leakage properties of cerasomes, and are unable to achieve combined delivery and sequential controlled release of chemotherapeutic drugs and immune checkpoint inhibitors [58, 59, 60, 61].

**SCHEME 1:**
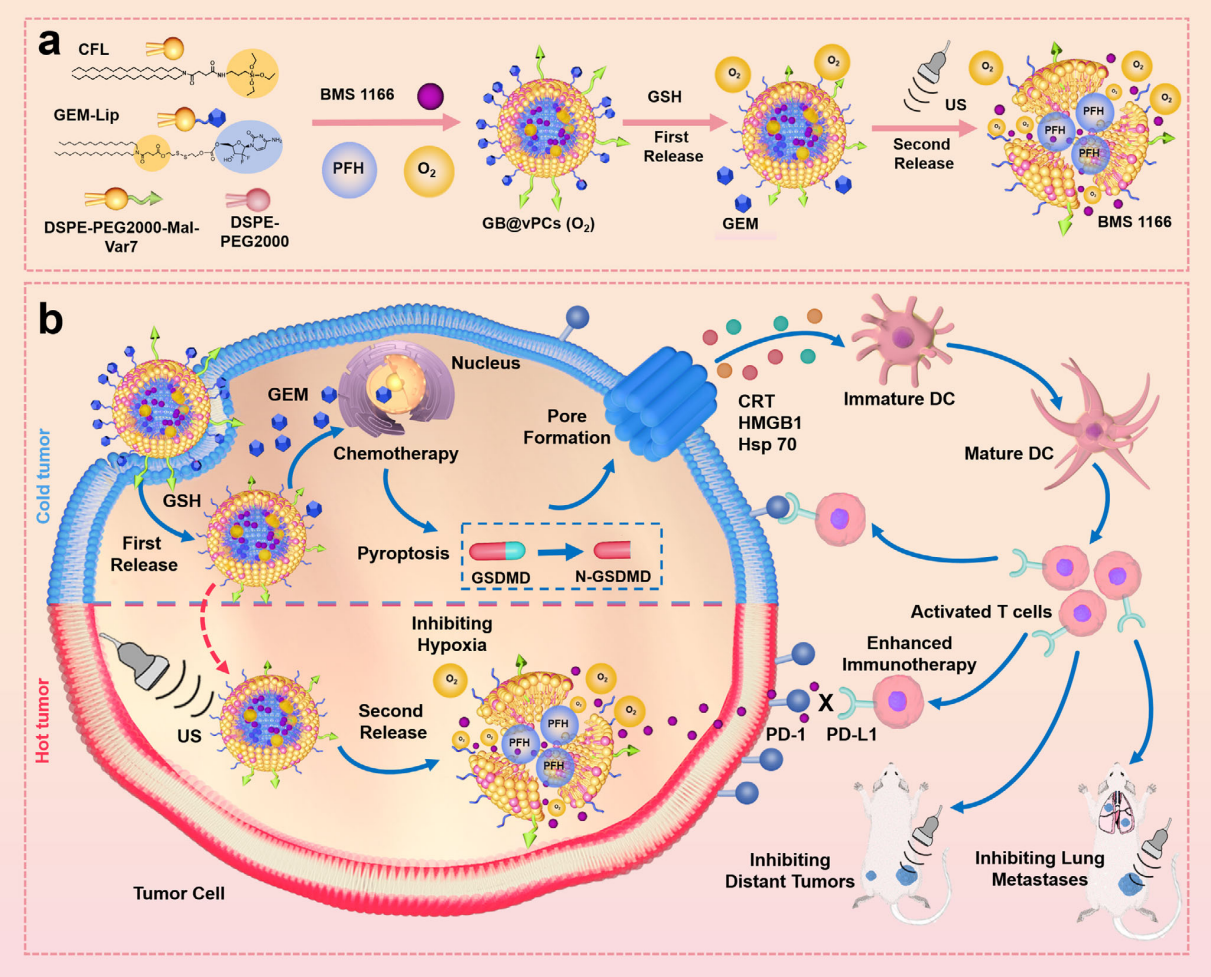
(a) Schematic illustration of the preparation of ultrasound/glutathione dual‐stimuli responsive GB@vPCs (O_2_) and their drug release triggered by either glutathione or ultrasound stimuli; (b) Schematic representation of the dual‐stimuli responsive GB@vPCs (O_2_) under a specific stimulation sequence, that is, first exposure to glutathione followed by ultrasound stimulation, sequentially releasing chemotherapeutic drugs and immune checkpoint inhibitor, accompanied by oxygen release to enhance the synergistic therapeutic effect.

Herein, we develop a refined therapeutic strategy using a dual responsive liposomal nanomedicine to control the action timing of chemotherapy drug and ICIs for achieving robust immunotherapy (Scheme 1). BMS1166 and gemcitabine (GEM) are selected as the model ICIs and chemotherapy drugs, respectively, where BMS1166 is a small‐molecule PD‐L1 inhibitor and GEM can trigger effective immune response. To ensure the consistent pharmacokinetic and biodistribution behaviors, BMS1166 and GEM are co‐assembly into one liposomal nanocarrier. More importantly, an appropriate release sequence may support combination chemoimmunotherapy: GEM is intended to be released first to induce immunogenic cell death and increase tumor immunogenicity, while the associated upregulation of PD‐L1 provides a rationale for subsequent BMS1166‐mediated inhibition of the PD‐1–PD‐L1 interaction. Based on this regard, BMS1166 and GEM can be designed to be physically loaded and chemically conjugated into nanoparticles, respectively, to achieve sequential drug release. And the specific physiological characteristics of tumor cells with high expression of GSH and Ultrasound‐induced cavitation effect are leveraged to tailor the on‐demand drug release behaviours. A lipid–GEM conjugate with disulfide bonds is synthesized, which can assemble with silane precursor containing cerasome‐forming lipid (CFL) and acid targeting lipid‐Var7 to form stable and active targeting liposomal nanoparticles and physically encapsulate BMS1166 and perfluorohexane (PFH) to obtain GB@vPCs NPs.

GB@vPCs with silicate framework surface has superior stability over liposomal drug, plus its active targeting can hold the capability to stably deliver drugs and retain long‐time at the target tumor area, facilitating the later control release of two drugs separately. After uptake by tumor cells, GB@vPCs rapidly release GEM by a GSH‐triggered disulfide bond breakage. The released GEM acts on tumor cells to induce ICD, pyroptosis and also upregulation of PD‐L1. BMS1166 encapsulated is highly stable and minimally leaks from GB@vPCs. When US is applied, the cavitation effect mediated by PFH leads to the release of BMS1166, which interferes with the PD‐1–PD‐L1 interaction, thus forming a good synergistic effect with the immune response triggered by chemotherapy drugs. Such a dual responsive nanoplatform holds a more potent effect on activating immunotherapy. In addition, the released oxygen in PFH can relieve tumor hypoxia by inhibiting hypoxia‐inducible factor‐1α (HIF‐1α) to enhance both the chemotherapy and immunotherapy. We show that GB@vPCs effectively inhibit primary and distant tumor growth, relapse and metastasis while form a strong immune memory in the mouse model.

## 2 Results and Discussion

### 2.1 Preparation and Formulation Optimization of GB@vPCs

**FIGURE 1:**
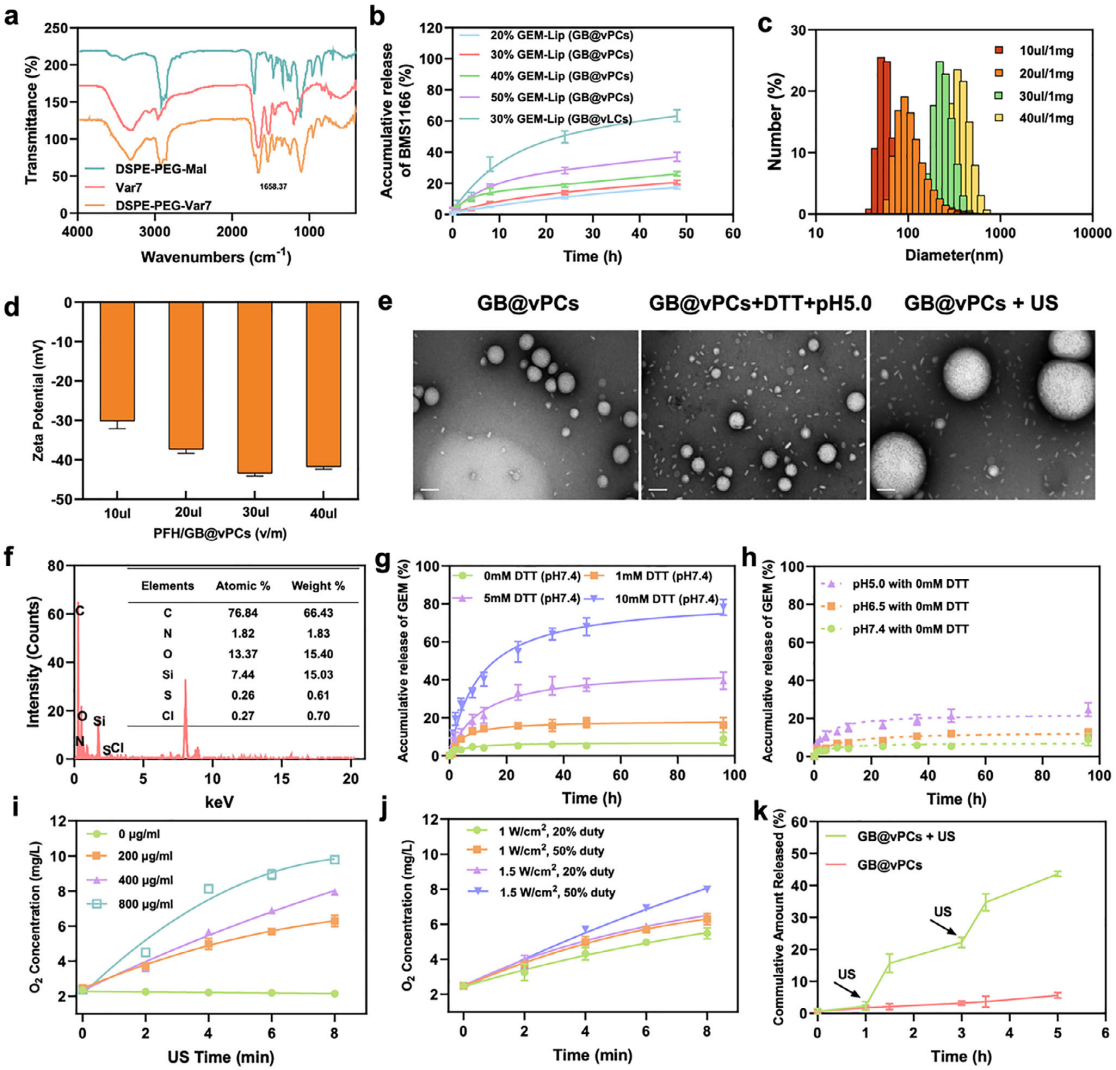
Synthesis and Characterization of GB@vPCs. (a) Fourier infrared spectra of DSPE‐PEG‐Mal, Var7, and DSPE‐PEG‐Var7. (b) Release profiles of BMS1166 from GB@vPLs and GB@vPCs with varying composition ratios. (c) The hydrated particle size of GB@vPCs with differing volumes of PFH. (d) The Zeta potential of GB@vPCs with differing volumes of PFH. (e) TEM images of GB@vPCs before and after incubation with 10 mM DTT at pH 5.0 for 12 h, and post‐ultrasound exposure (1.0 MHz, 1 W cm^−2^, 50% duty cycle, 5 min) (scale bar: 100 nm). (f) EDS spectrum of GB@vPCs and the atomic and weight percentage of various elements. (g) GEM release curves from G@vPCs under varying DTT concentrations. (h) GEM release curves from G@vPCs at different pH levels. (i) Oxygen release profiles of GB@vPCs(O_2_) at various concentrations under ultrasound stimulation (1.0 MHz, 1 W cm^−2^, 50% duty cycle). (j) Oxygen release profiles of 200 µg mL^−1^ GB@vPCs(O_2_) under varying ultrasound parameters. (k) BMS1166 release profiles from GB@vPCs with and without ultrasound irradiation (1.0 MHz, 1 W cm^−2^, 50% duty cycle).

To prepare the GSH‐responsive cerasome, a gemcitabine prodrug lipid (GEM‐Lip) with a disulfide bond was successfully synthesized through two‐step chemistry (Figure S1), validated by TOF mass spectrometry (Figures S2 and S3). Given the acidic tumor microenvironment, the pH‐sensitive Var7 peptide was incorporated to enhance tumor targeting. Covalent linkage of Var7 to DSPE‐PEG2000‐maleimide (DSPE‐PEG2000‐Mal) yielded DSPE‐PEG2000‐Var7, confirmed by Fourier transform infrared spectroscopy (FTIR) showing peaks at 1658 cm^−1^ (amide bond) and 2916 cm^−1^ (PEG C‐H stretch) (Figure 1a), and mass spectrometry indicating a MW of ≈5959 Da (Figure S4), aligning with the expected sum of DSPE‐PEG‐Mal (≈2900 Da) and Var7 peptide (≈3065 Da).

GB@vPCs were fabricated in several steps: GEM‐Lip, CFL, DSPE‐PEG2000, and DSPE‐PEG2000‐Var7 were dissolved separately in solvents, mixed in a defined molar ratio, with BMS1166 added before sonication into deionized water to form GB@PCs. Subsequently, PFH was then incorporated through probe sonication to yield GB@vPCs. The prepared GB@vPCs were allowed to incubate at room temperature for 24 h to facilitate condensation of surface silanol groups, forming a stable silicate network structure. Finally, the GB@vPCs were exposed to an oxygen‐saturated chamber for a duration of 60 min to facilitate oxygen loading, yielding O_2_‐saturated GB@vPCs (O_2_). To investigate the optimal molar ratio of GEM‐Lip to CFL for cerasome stability, GB@vPCs variants with varied molar compositions (20:65, 30:55, 40:45, 50:35) were prepared (Table S1) and analyzed for BMS1166 release profiles (Figure 1b). As a control, conventional liposomes (GB@vPLs) devoid of silicate components were prepared using DSPC instead of CFL (Table S1). As shown in Figure 1b, with increasing CFL molar ratio and decreasing GEM‐Lip molar ratio, the amount of BMS1166 released from GB@vPCs systems decreased, attributed to the strengthened silicate layer and thus, enhanced cerasome stability. At 55% CFL, drug retention was comparable to that observed at 65%, with GB@vPCs releasing less than 20% of BMS1166 within 48 h. In contrast, GB@vPLs with 55% DSPC released approximately 63% of the drug over the same period, highlighting cerasomes' superiority in stability. Based on these insights, a 30:55 GEM‐Lip:CFL ratio was determined as the optimal balance for both stability and GEM loading capacity.

In the optimization of GB@vPCs synthesis, the BMS1166 feeding ratio was adjusted to enhance encapsulation efficacy. By incrementally increasing the BMS1166 feeding ratio during formulation, a series of GB@vPCs samples were prepared. Purification ensued to remove unencapsulated BMS1166, followed by high‐performance liquid chromatography (HPLC) analysis to quantify encapsulation efficiency and BMS1166 loading in each sample. As shown in Figure S5, with increasing BMS1166 feeding ratio, the encapsulation efficiency showed a gradual decline, whereas drug loading correspondingly increased. When the mass feeding ratio reached 1:5, the drug loading content nearly reached saturation, with an encapsulation efficiency of 64.38 ± 4.15% and a loading content of 11.41 ± 0.65%. Consequently, this ratio was deemed optimal for achieving balanced loading efficiency and drug payload. Similarly, the PFH dosage in GB@vPCs fabrication was optimized. Varying volumes of PFH (10 µL to 40 µL per mg of nanoparticles) were introduced to pre‐formed GB@PCs, yielding GB@vPCs with distinct PFH contents and particle sizes. As shown in Figure 1c, PFH addition correlated positively with particle swelling, expanding sizes from 87.39 ± 15.87 to 358.05 ± 30.30 nm. All formulations exhibited negative Zeta potentials (Figure 1d), ensuring colloidal stability and preventing aggregation. Balancing tumor penetration potential of smaller particles with necessary PFH capacity for oxygen transport, a PFH addition level of 20 µL per mg nanoparticle was established as optimal.

The colloidal stability of GB@vPCs was evaluated at 37°C over 7 days. DLS analysis showed minimal changes in particle size, zeta potential, and PDI (Figure S6), the hydrodynamic diameter remained within 101–118 nm over 7 days, and the PDI remained around 0.3, indicating no aggregation. These results confirm the excellent short‐term stability of GB@vPCs under physiological conditions, supporting their in vivo application.

### 2.2 Characterization of GB@vPCs

Transmission electron microscopy (TEM) analysis highlighted the exceptional dispersal properties of GB@vPCs, exhibiting dry‐state particle diameters averaging 90 nm (Figure 1e), marginally less than their hydrated counterparts measuring 119.13 ± 5.44 nm, and the PDI was maintained at around 0.3 (Figure 1c). This discrepancy arises from the formation of a hydration layer as nanoparticles absorb water in solution, inflating their dimensions compared to TEM observations in a desiccated state. After ultrasound treatment, DLS revealed a marked size increase to 550.03 ± 26.63 nm (Figure S7), consistent with the cavitation‐induced morphological changes observed by TEM. The silicate shell simultaneously fulfills three functional requirements through its structural design. Before ultrasound, the ordered mesoporous network provides a rigid, leak‑proof storage structure, as evidenced by low‑level BMS1166 leakage over 48 h without ultrasound (Figure 1b). Upon ultrasound irradiation, PFH vaporization is thought to generate localized mechanical stress that transiently disrupts the ordered lattice, thereby promoting payload release. Formation of the silicate network was corroborated by Fourier transform infrared spectroscopy (FTIR), revealing distinctive peaks at 1100 and 950 cm^−1^ in GB@vPCs and Cerasome spectra (Figure S8), indicative of Si─O─Si and Si─OH bond vibrations, confirming the presence of a silicate coating. Energy‐dispersive X‐ray spectroscopy (EDS) further validated silicon incorporation in GB@vPCs (Figure 1f). Notably, GB@vPCs retained a spherical morphology under 10 mM DTT and acidic conditions, testifying to their robustness. Nevertheless, exposure to US resulted in particle diameter expansion, attributed to PFH vaporization‐induced volume increase (Figure 1e). This phenomenon was positively relevant for promoting the release of the encapsulated drugs within GB@vPCs. The core–shell boundary was not clearly resolved by TEM, likely due to the low electron density contrast between the PFH core and the lipid shell.

Upon internalization into cells, GB@vPCs facilitated GEM release through disulfide cleavage by glutathione. Given glutathione's instability, DTT was employed to simulate GEM release dynamics at various concentrations. As shown in Figure 1g, GEM release exhibited a strong, concentration‐dependent response to DTT, which mimics the high GSH levels present in the tumor microenvironment. In contrast, the effect of pH on drug release was minimal. As shown in Figure 1h, although a slight increase in drug release was observed at lower pH values, this increase was negligible compared with the pronounced release induced by DTT. Thus, in our system, the pH sensitivity is primarily intended to promote particle accumulation in the acidic tumor microenvironment, whereas the actual drug release is predominantly governed by redox‐sensitive disulfide bond cleavage. Furthermore, US‐mediated oxygen release from GB@vPCs (O_2_) was systematically studied (Figure 1i,j), demonstrating a direct correlation between release quantity and US duration/intensity, as well as GB@vPCs (O_2_) concentration. Adjusting US parameters, specifically power and duty cycle, resulted in tunable oxygen output, highlighting the precision of US in controlling release kinetics. Additionally, the study probed the release behavior of the drug BMS1166 under US stimulation (Figure 1k), showing negligible BMS1166 release in the absence of US. Conversely, upon US application, a substantial escalation in BMS1166 release was detected. Collectively, these outcomes offer robust evidence affirming US's efficacy in facilitating drug release processes.

### 2.3 Cell Uptake and Acidic Microenvironment Targeting Ability of GB@vPCs

**FIGURE 2:**
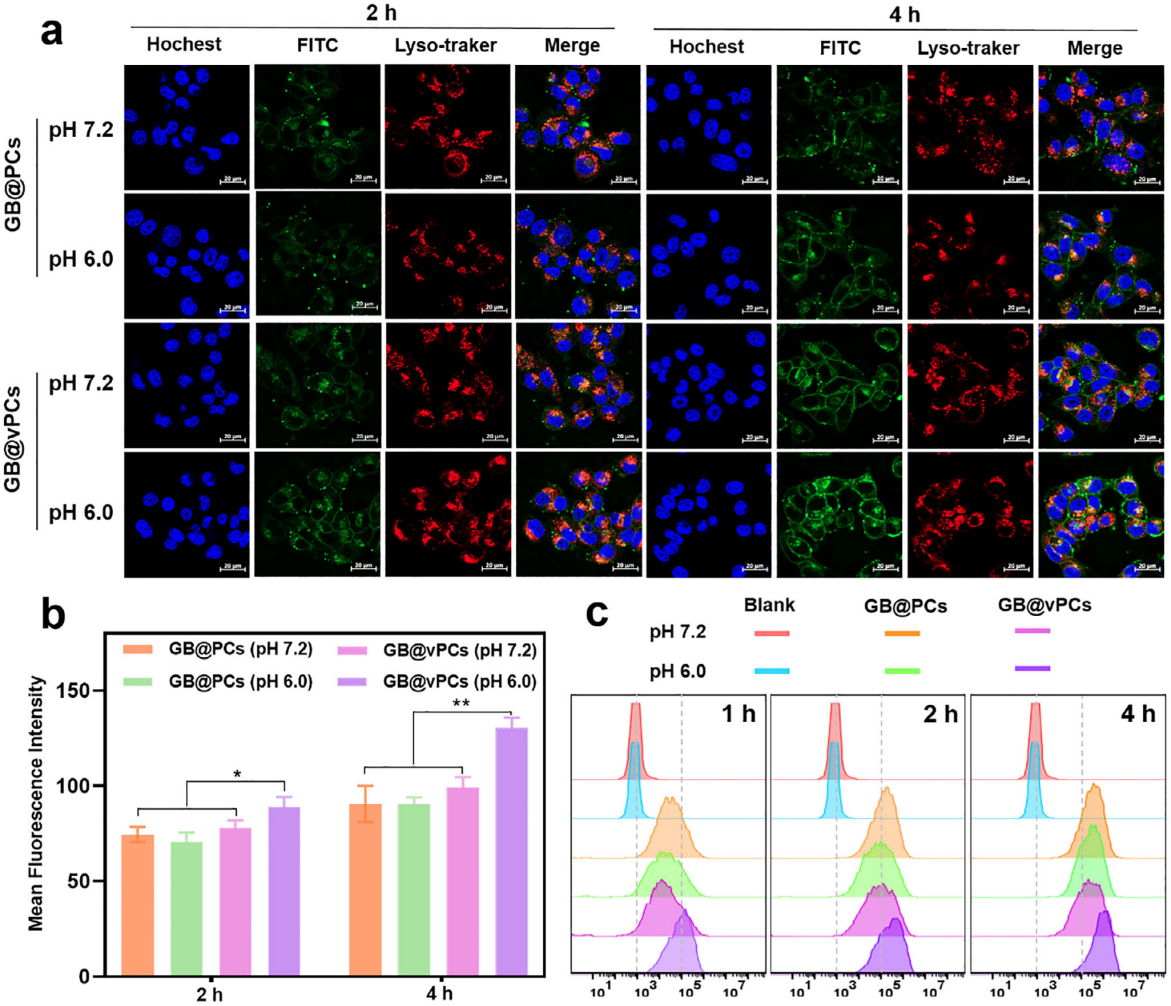
Cell uptake and acidic microenvironment targeting ability of GB@vPCs. (a) CLSM images of GB@PCs and GB@vPCs internalization after 2 and 4 h of incubation at pH 7.2 and pH 6.0, respectively (Scale bar: 20 µm). (b) Quantification of mean fluorescence intensity from panel (a). Data are presented as mean ± SD (n = 3). (c) Flow cytometry analysis of 4T1 cells after incubation with GB@PCs and GB@vPCs for 1, 2, and 4 h at pH 7.2 and pH 6.0.

To examine the tumor‐targeting efficacy of GB@vPCs under acidic conditions, GB@PCs and GB@vPCs were labeled with FITC and incubated with 4T1 cells at both neutral (pH 7.2) and simulated tumor acidic microenvironment (pH 6.0) for varying durations. Following this, cellular internalization was examined using confocal laser scanning microscopy (CLSM) and flow cytometry. As shown in Figure 2a, the intracellular fluorescence of both GB@vPCs and GB@PCs increased over time, evidencing a marked time dependency in the cellular uptake of these nanoparticles. Notably, under neutral conditions, the rate of fluorescence intensity increase within cells was similar for GB@vPCs and GB@PCs, indicating no substantial difference in cellular uptake between the two formulations. Conversely, in acidic conditions, GB@vPCs displayed heightened tumor‐targeting capabilities. Quantitative analysis (Figure 2b) revealed that GB@vPCs were internalized at a level 1.3‐fold greater than GB@PCs under acidic conditions, highlighting the substantial role of the Var7 peptide moiety in enhancing the GB@vPCs' targeting efficiency within the acidic tumor microenvironment. Consistent with this, flow cytometry data (Figure 2c) showed a sustained elevation in intracellular fluorescence intensity of GB@vPCs at different time points under acidic conditions, with a more pronounced increase compared to GB@PCs, affirmatively validating the significant boost in nanoparticle targeting conferred by the Var7 peptide specifically in acidic environments.

### 2.4 Induction of Immunogenic Phenotype in Tumor Cells by GB@vPCs

**FIGURE 3:**
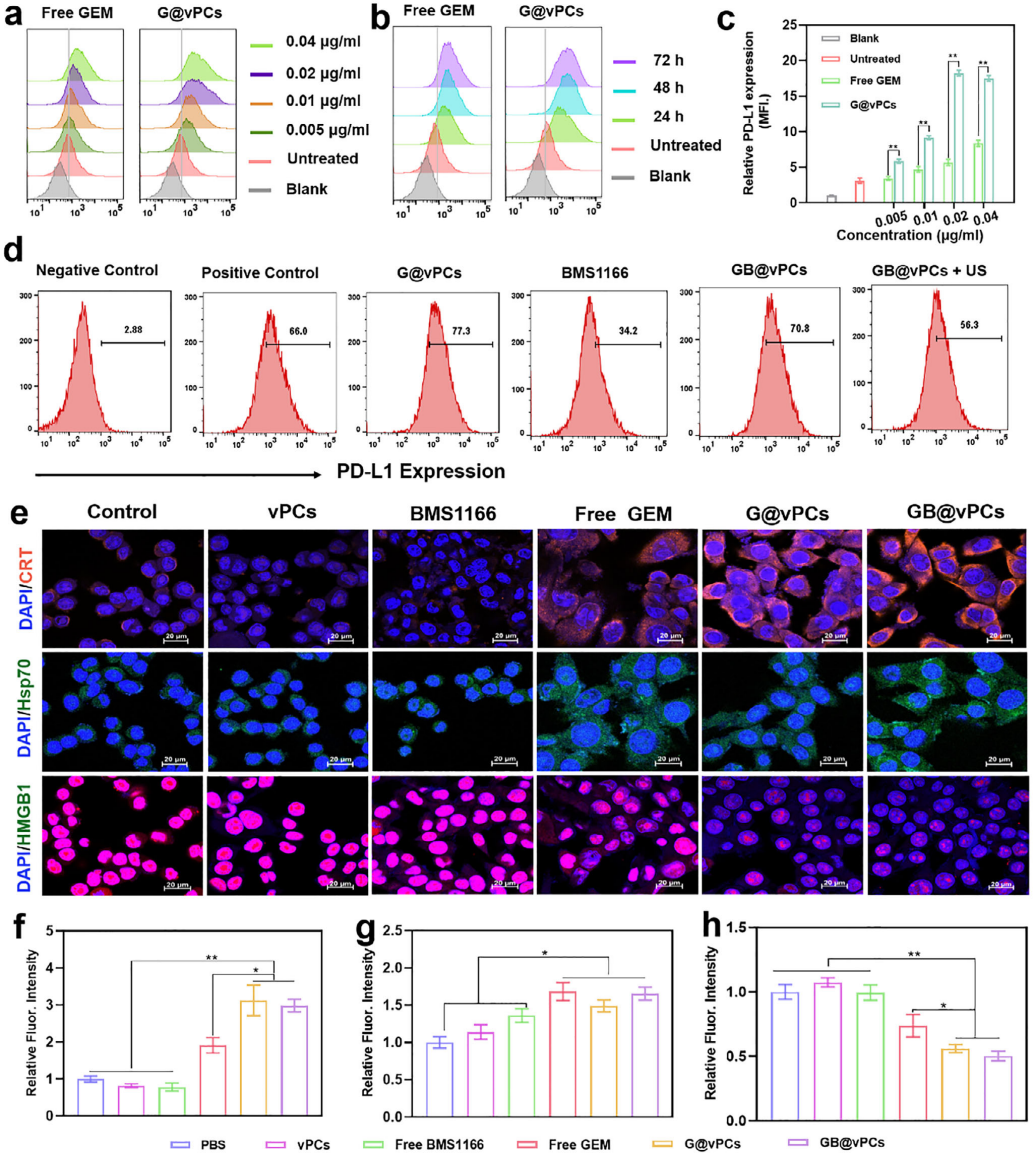
Induction of Immunogenic Phenotype by GB@vPCs. (a) Flow cytometry of PD‐L1 expression on 4T1 cells after 24 h treatments with varying concentrations of Free GEM and G@vPCs, and (b) PD‐L1 expression on 4T1 cells treated with a fixed concentration (0.04 µg mL^−1^) for differing durations; (c) quantification of flow cytometry from (a), presented as mean ± SD (n = 3); (d) flow cytometry of surface PD‐L1 immunostaining on 4T1 cells following various treatments. (e) CLSM images of surface CRT and Hsp70 expression, and loss of nuclear HMGB1 fluorescence in 4T1 cells after different treatments. (f) Quantification of relative surface CRT fluorescence intensity in 4T1 cells from (e), presented as mean ± SD (n = 3). (g) Quantification of relative surface Hsp70 fluorescence intensity in 4T1 cells from (e), presented as mean ± SD (n = 3). (h) Quantification of relative nuclear HMGB1 fluorescence intensity in 4T1 cells from (e), presented as mean ± SD (n = 3).

Chemotherapeutics have the capacity to evoke an immunogenic transformation in tumor cells, often paralleled by increased surface PD‐L1 expression. To explore GB@vPCs' potential in facilitating such immunomodulatory shifts, free GEM and G@vPCs were co‐incubated with 4T1 cells across a concentration gradient ranging from 0.005 µg/mL to 0.04 µg/mL, with variable incubation spans of 24 to 72 h. Surface PD‐L1 expression dynamics were examined using flow cytometry. As shown in Figure 3a,c, increasing drug concentrations led to a gradual rise in PD‐L1 expression on the surface of 4T1 tumor cells. At a stable concentration of 0.04 µg mL^−1^, PD‐L1 expression progressively increased over time, demonstrating a clear time‐dependent increase (Figures 3b and S9). Notably, GEM conjugated in G@vPCs induced a more pronounced upregulation of PD‐L1 compared to free GEM under equivalent conditions. Specifically, at a concentration of 0.04 µg mL^−1^ and after 24 h of incubation, PD‐L1 expression induced by G@vPCs was three‐fold higher than that caused by free GEM. These findings imply that G@vPCs could efficiently deliver drugs into tumor cells via endocytosis, potentially yielding enhanced therapeutic outcomes and immunomodulatory effects compared to freely diffusing drugs.

To evaluate GB@vPCs' efficacy in blocking tumor cell immune checkpoint function, GB@vPCs were co‐cultured with 4T1 cells, followed by flow cytometry analysis of PD‐L1 expression on the cell surface. As shown in Figure 3d, G@vPC‐treated cells presented higher PD‐L1 levels than the positive control, a reflection of chemotherapy's PD‐L1‐elevating effect. By contrast, cells exposed to free BMS1166 showed a drastic reduction in PD‐L1 expression, declining from 66% in the control to 34.2%, affirming BMS1166's potency in suppressing the PD‐1/PD‐L1 pathway and enhancing cytotoxic T‐cell's tumor targeting. Without US exposure, BMS1166 release from GB@vPCs was distantly inadequate to suppress PD‐L1. Conversely, US exposure activated a substantial BMS1166 release, notably diminishing PD‐L1 levels, confirming BMS1166's highly effective immune checkpoint inhibition. This action interferes with the PD‐1/PD‐L1 signaling pathway, enhancing the immune system's recognition and attack on tumor cells.

Given the design of cerasomes intended to first stimulate the immune system via chemotherapy, the effectiveness of GB@vPCs in inducing immunogenic cell death (ICD) in tumor cells was evaluated. ICD involves the release of damage‐associated molecular patterns (DAMPs), including calreticulin (CRT), high mobility group box 1 protein (HMGB1), and surface‐expressed 70 kDa heat shock protein (HSP70), which could activate dendritic cells (DCs) and boost anti‐tumor immune responses. To quantify this, CRT surface exposure was analyzed by flow cytometry. As seen in Figure S10, neither PBS nor vPCs‐only controls showed a significant increase in CRT expression. However, treatment with free GEM, G@vPCs, and GB@vPCs resulted in ascending CRT expression levels, reaching 9.27%, 19.3%, and 20.8%, respectively, CRT expression was significantly higher in the GB@vPCs group compared with free GEM (*p* = 0.0022). To further validate these findings, CLSM was used to examine CRT surface exposure, Hsp70 surface expression, and loss of nuclear HMGB1 fluorescence. As shown in Figure 3e–h, compared with PBS‐, vPCs‐, and free BMS1166‐treated cells, cells treated with free GEM, G@vPCs, and GB@vPCs showed increased CRT surface expression and Hsp70 expression, together with reduced nuclear HMGB1 fluorescence, strongly affirming GEM's ability to induce ICD and suggesting that nanoencapsulation of GEM enhances its ICD‐inducing potential, facilitating a more robust immune response in vivo.

### 2.5 Impact of GB@vPCs on Hypoxia, Drug Resistance, and Invasion of Cells

**FIGURE 4:**
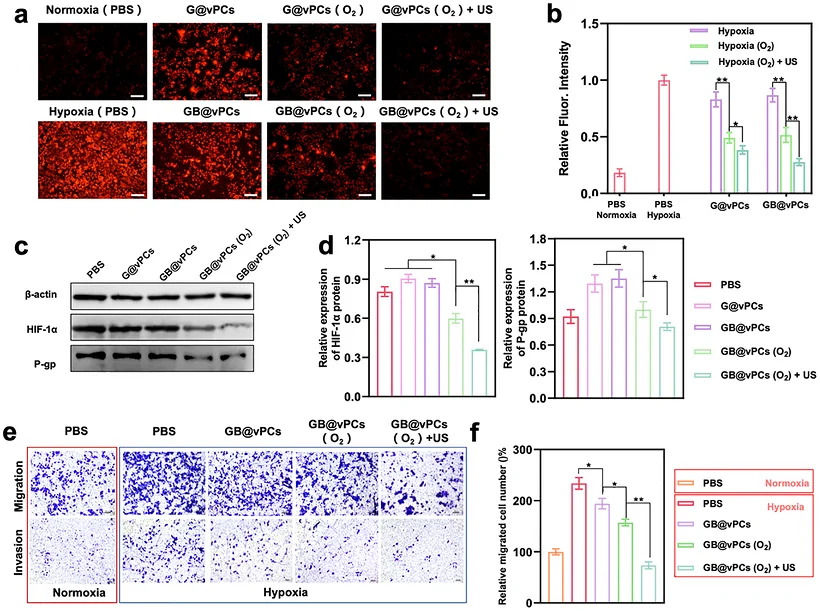
Impact of GB@vPCs on hypoxia, drug resistance, and invasion of cells. (a) Fluorescence microscopy images of [Ru(dpp)_3_]Cl_2_ in 4T1 cells following various treatments (Scale bar: 100 µm). (b) Quantitative analysis of [Ru(dpp)_3_]Cl_2_ fluorescence intensity within 4T1 cells under different treatments, data represented as mean ± SD (n = 3). (c) Western blot analysis of HIF‐1α and P‐gp expression in 4T1 cells after different treatments. (d) Quantification of HIF‐1α and P‐gp protein expression from (c), data are expressed as mean ± SD (n = 3). (e) Evaluation of migration and invasion capacities of 4T1 cells under different treatments using the Transwell assay. (f) Quantification of migration capacity from (e), data represented as mean ± SD (n = 3).

To investigate whether GB@vPCs loaded with oxygen could effectively alleviate hypoxia in tumor cells, the oxygen probe tris(4,7‐diphenyl‐1,10‐phenanthroline)ruthenium(II) dichloride ([Ru(dpp)_3_]Cl_2_) was employed. This probe exhibits distinct optical properties under different oxygen concentrations: weak fluorescence in high‐oxygen environments and strong red fluorescence under low‐oxygen conditions. Initially, 4T1 cells were cultured under 1% oxygen to mimic actual tumor hypoxia. Post‐treatment with various formulations, cells were examined under a fluorescence microscope. As shown in Figure 4a,b, bright red fluorescence was observed in PBS, G@vPCs, and GB@vPCs groups, indicating severe hypoxia. Conversely, treatment with oxygen‐saturated G@vPCs (O_2_) and GB@vPCs (O_2_) led to a marked reduction in red fluorescence, verifying their oxygen‐releasing capability and effective relief of cellular hypoxia. Strikingly, upon combining oxygen‐saturated GB@vPCs (O_2_) with US, the red fluorescence was nearly abolished, highlighting ultrasound's capacity to accelerate oxygen release, thereby dramatically improving cellular hypoxia.

It is well‐established that hypoxia induces upregulation of the HIF‐1α protein, which in turn activates transcription of the multidrug resistance gene 1 (MDR1). Consequently, expression of P‐glycoprotein (P‐gp), encoded by MDR1, increases, actively extruding chemotherapeutics from tumor cells and promoting multidrug resistance (MDR). To explore the changes in HIF‐1α and P‐gp expression at the cellular level, Western blot analysis was performed. As shown in Figure 4c,d, under hypoxic conditions, the expression levels of HIF‐1α in the PBS control group, G@vPCs group, and GB@vPCs group remained comparable. However, following treatment with oxygen‐loaded GB@vPCs (O_2_), a marked reduction in protein expression was observed, with the GB@vPCs (O_2_) + US group, which received additional US treatment, exhibiting the lowest expression, strongly validating the efficacy of oxygen‐carrying GB@vPCs (O_2_) in alleviating cellular hypoxia. For the P‐gp protein, its expression was upregulated after treatment with G@vPCs and GB@vPCs. Conversely, upon treatment with GB@vPCs (O_2_), a decline in P‐gp expression was noted, and this downregulation was further accentuated in the GB@vPCs (O_2_) + US group, indicating that oxygenated GB@vPCs (O_2_) effectively suppress P‐gp expression, thereby contributing to alleviating multidrug resistance in tumors.

Given the direct correlation between HIF‐1α overexpression and increased migration and invasive capabilities of tumor cells, Transwell assays were employed to evaluate the migration and invasion abilities of 4T1 cells post‐treatment. As shown in Figures 4e,f and S11, hypoxic 4T1 cells showed increased migration and invasive capabilities compared to normoxic cells, highlighting the crucial role of hypoxia in tumor progression. While GB@vPCs treatment slightly reduced migration and invasion, it failed to fully counteract hypoxia‐induced effects. However, treatment with oxygen‐loaded GB@vPCs (O_2_) led to a more substantial decrease in invasive capacity. Critically, combining GB@vPCs (O_2_) with US reduced both migration and invasion to the lowest levels, emphasizing the importance of oxygen delivery in alleviating intrinsic hypoxia and suppressing malignant characteristics. These findings validate that delivering oxygen via GB@vPCs (O_2_), particularly with US, effectively mitigates tumor microenvironment hypoxia, significantly reducing the migratory and invasive potential of tumor cells.

### 2.6 Cytotoxic Effects of GB@vPCs

**FIGURE 5:**
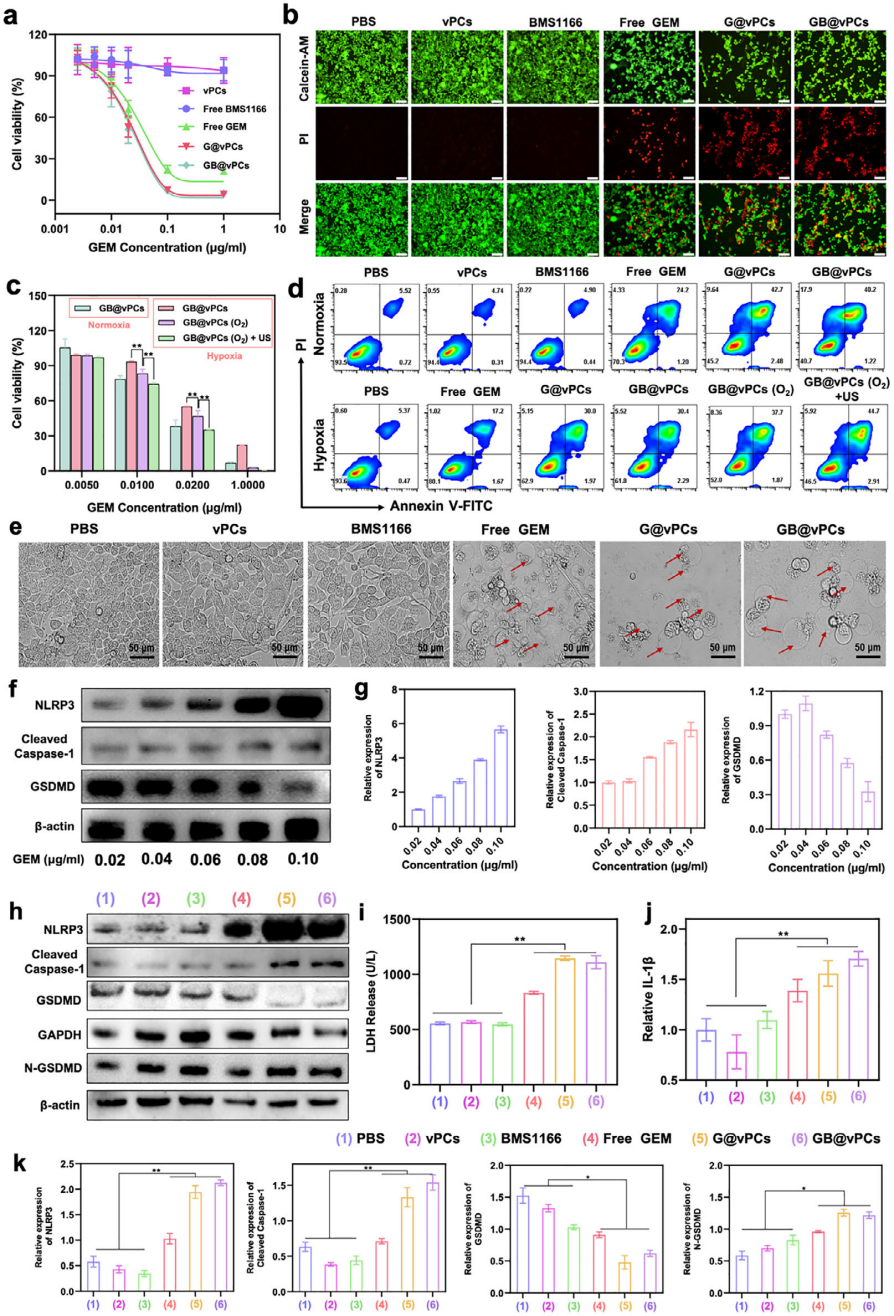
Cytotoxic Effects of GB@vPCs on 4T1 Cells. (a) Viability of 4T1 cells after 24 h incubation with various drugs, determined by the CCK‐8 assay. (b) Fluorescence microscopy images of 4T1 cells stained with Calcein AM/PI after 24 h incubation with different treatments (scale bar: 100 µm). (c) Viability of 4T1 cells under hypoxic conditions after 24 h incubation with various treatments, determined by the CCK‐8 assay. (d) Apoptosis levels of 4T1 cells under normoxic and hypoxic conditions after 24 h incubation with different drugs with or without US, measured using the Annexin V/PI apoptosis detection kit. (e) Microscopic images of 4T1 cells after various treatments (scale bar: 50 µm). (f) Western blot analysis of NLRP3, Cleaved caspase‐1, and GSDMD protein expression in 4T1 cells treated with varying concentrations of GEM. (g) Quantification of NLRP3, Cleaved caspase‐1, and GSDMD protein expression levels from (f). (h) Western blot analysis of NLRP3, Cleaved caspase‐1, GSDMD, and N‐GSDMD protein expression in 4T1 cells after different treatments. (i) LDH release from 4T1 cells after various treatments. (j) Secretion of IL‐1β from 4T1 cells after different treatments. (k) Quantification of NLRP3, Cleaved caspase‐1, GSDMD, and N‐GSDMD protein expression levels from (h).

The cytotoxic efficacy of GB@vPCs on cells was subsequently investigated. First, the cytotoxic effects of different formulations and drug concentrations on 4T1 cells were assessed under normoxic conditions. As shown in Figure 5a, neither the carrier vPCs nor free BMS1166 at various concentrations caused noticeable cell damage after 24 h of incubation, affirming the safety of the drug carrier and the non‐toxic nature of free BMS1166 to cells. In contrast, free GEM, G@vPCs, and GB@vPCs all demonstrated dose‐dependent cytotoxicity, attributed to GSH‐mediated cleavage of disulfide bonds in GEM‐Lip within tumor cells, releasing GEM to exert chemotherapeutic toxicity. Remarkably, G@vPCs and GB@vPCs exhibited increased cytotoxicity compared to free GEM, potentially due to increased drug uptake by tumor cells through nanoparticle endocytosis. Cells were co‐stained with Calcein‐AM/PI to further visualize the effects of different treatments. As shown in Figure 5b, cells treated with G@vPCs and GB@vPCs displayed higher red fluorescence (indicative of dead cells) and lower green fluorescence (indicative of live cells) compared to free GEM, highlighting their superior tumor‐killing capacity. Subsequently, Annexin V‐FITC/PI staining was employed to assess apoptosis rates post‐treatment. As shown in Figure 5d, vPCs and BMS1166 treatment resulted in only 4.79 ± 0.06% and 4.93 ± 0.03% apoptotic 4T1 cells, respectively, reinforcing the safety of the carrier and the non‐toxicity of BMS1166. Treatment with free GEM, G@vPCs, and GB@vPCs led to approximately 20.27 ± 6.39%, 44.43 ± 2.19%, and 41.7 ± 7.37% apoptosis, respectively, consistent with Figure 5a,b.

Subsequently, the oxygen‐loaded and non‐oxygen‐loaded nanoparticles were compared to validate the influence of oxygen on chemotherapy. 4T1 cells were cultured under 1% O_2_ hypoxia, and cytotoxicity was evaluated using the CCK‐8 assay. As seen in Figure 5c, GB@vPCs (O_2_) showed greater cytotoxicity than GB@vPCs after 24 h of incubation, which was further augmented with the addition of US exposure, exhibiting toxic effects consistent with those under normoxic conditions. Notably, vPCs + US did not cause significant cell damage, excluding any inherent toxicity from US (Figure S12). Annexin V‐FITC/PI staining further confirmed that free GEM, G@vPCs, and GB@vPCs had reduced toxicity on hypoxia‐cultured cells compared to normoxia, suggesting that relieving hypoxia enhances chemotherapy's cytotoxic effect. Specifically, GB@vPCs (O_2_) enhanced the cytotoxicity, suggesting that oxygen release augments chemotherapy's killing efficiency. Moreover, US further amplified this effect, indicating its role in triggering rapid O_2_ release from GB@vPCs, significantly alleviating cellular hypoxia and intensifying chemotherapy (Figures 5d and S13). Future studies incorporating a triple co‐culture system of tumor cells, dendritic cells, and CD8^+^ T lymphocytes will be valuable to further evaluate the in vitro cell‐killing effect and to dissect the antigen‐presentation cascade underlying the observed anti‐tumor immunity.

Given that chemotherapy can also induce pyroptosis, a highly inflammatory form of programmed cell death, we examined cell morphology for signs of pyroptosis. As shown in Figure 5e, vPCs and BMS1166 treatments did not alter cell morphology, resembling the PBS control. Free GEM, G@vPCs, and GB@vPCs induced typical pyroptotic features, including large bubble‐like protrusions (indicated by red arrows), and reduced cell density, which are features indicative of pyroptotic cell death. Pyroptosis involves NLRP3 inflammasome activation, leading to caspase‐1 activation and cleavage of GSDMD, which forms pores in the cell membrane and facilitates inflammatory content release. The impact of different GEM concentrations on NLRP3, Cleaved caspase‐1, and GSDMD was investigated. As shown in Figure 5f,g, as GEM concentration raised, NLRP3 and Cleaved caspase‐1 expression increased while GSDMD decreased, confirming GEM‐induced pyroptosis with NLRP3 upregulation and GSDMD cleavage. Following GB@vPCs treatment, NLRP3, Cleaved caspase‐1, and N‐GSDMD expression were significantly upregulated, with GSDMD downregulated, to approximately 2.0‐, 2.2‐, and 1.3‐fold relative to GEM alone, respectively (Figure 5h,k). These changes, together with the observed morphological alterations, are consistent with enhanced pyroptosis pathway engagement, potentially attributable to increased intracellular GEM accumulation via targeted delivery. Lactate dehydrogenase (LDH) release and interleukin‐1β (IL‐1β) secretion were assessed as markers of cell membrane rupture and pyroptosis, respectively. As shown in Figure 5i,j, G@vPCs and GB@vPCs treatments led to a significant increase in LDH release and IL‐1β secretion, indicating efficient induction of pyroptosis by GB@vPCs. Collectively, these results suggest that GB@vPCs can augment GEM‐induced pyroptotic pathway activation, which may contribute to an immunostimulatory tumor microenvironment favorable for subsequent immunotherapy.

Notably, the magnitude of N‐GSDMD upregulation was weaker than that of the upstream signaling molecules. It may reflect the inherent biological behavior of GSDMD‐N as a membrane‐pore‐forming executor, which is rapidly degraded or cleared upon pore formation, leading to its relatively modest accumulation compared to upstream signaling components. Alternatively, it may indicate that under the current experimental conditions, pyroptosis is still in an early phase of activation. Future studies using genetic or pharmacological tools will therefore be essential to confirm the necessity of GSDMD in mediating the observed anti‐tumor and immune effects and to clarify the precise molecular mechanisms involved. Furthermore, while our data indicate activation of the NLRP3/caspase‐1/GSDMD pyroptosis pathway and associated immune effects, we note that the relative contributions of pyroptosis and apoptosis to the observed anti‐tumor immune activation were not independently quantified. Future studies incorporating specific pyroptosis and apoptosis inhibitors will be necessary to further dissect the respective roles of these two mechanisms.

### 2.7 In Vivo Ultrasound/Fluorescence Dual‐Modal Imaging Capabilities of GB@vPCs

Before in vivo experiments, the hemocompatibility of GB@vPCs(O_2_) was first evaluated through a hemolysis test, as shown in Figure S14a. The results indicated that red blood cells (RBCs) maintained their structural integrity without significant hemolysis upon co‐incubation with GB@vPCs(O_2_) across a range of concentrations from 31.3 to 500 µg mL^−1^, resembling the condition observed with phosphate‐buffered saline (PBS) as a negative control. Remarkably, even after a 4 h incubation at the maximum concentration of 500 µg mL^−1^, the hemolysis rate was only 8.25 ± 0.08%, strongly validating the excellent blood compatibility of GB@vPCs(O_2_). To further confirm the in vivo safety of GB@vPCs(O_2_), they were intravenously injected into mice, with blood routine tests performed before injection and on days 1, 7, 14, and 28 post‐injection. As shown in Figure S14b, compared to pre‐injection levels, no substantial changes were detected in the hematological parameters of mice at these time points, reasserting the safety of GB@vPCs for in vivo use.

**FIGURE 6:**
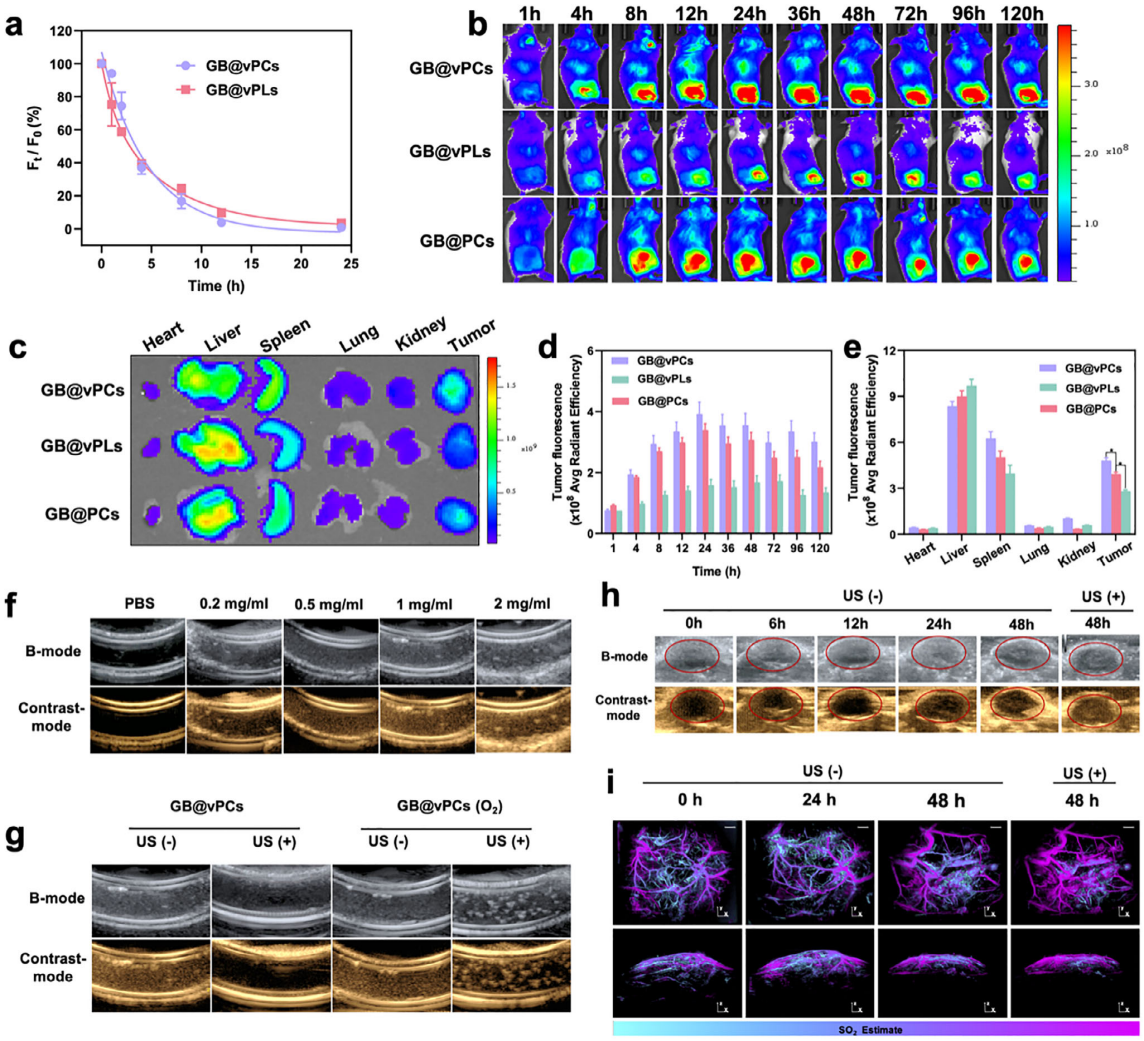
Multimodal ultrasound, photoacoustic and fluorescence imaging properties of GB@vPCs. (a) Pharmacokinetics of GB@vPCs and GB@vPLs in mouse plasma; (b) whole‐body in vivo fluorescence images after intravenous administration of GB@vPCs, GB@vPLs, and GB@PCs (GEM dose of 2.5 mg kg^−1^); (c) fluorescence images of major organs and tumors 120 h after intravenous injection of GB@vPCs, GB@vPLs, and GB@PCs; (d) quantitative analysis of fluorescence from (b); (e) quantification of fluorescence from (c); (f) in vitro ultrasound imaging of GB@vPCs at various concentrations; (g) in vitro ultrasound imaging of GB@vPCs and GB@vPCs(O_2_) with and without ultrasound irradiation (1 MHz, 1 W cm^−2^, 20% duty cycle, 5 min); (h) ultrasound imaging of mouse tumors at various time points post intravenous injection of GB@vPCs(O_2_), along with ultrasound imaging at 48 h following additional ultrasound exposure; (i) photoacoustic imaging of mice at different time points after GB@vPCs(O_2_) injection, including photoacoustic imaging at 48 h after supplementary ultrasound application.

Additionally, we investigated the pharmacokinetic properties of GB@vPCs. By labeling GB@vPCs and GB@vPLs with DSPE‐Cy5.5 and intravenously administering them to healthy mice, we monitored changes in Cy5.5 fluorescence intensity in plasma over different time intervals. Cy5.5 fluorescence was used as a semi‐quantitative tracer to monitor the time‐dependent relative retention of the labeled formulation in plasma. Because the fluorescence signal was not calibrated to absolute plasma concentration and the administered tracer dose was not quantified, conventional pharmacokinetic parameters were not derived. As shown in Figure 6a, compared to GB@vPCs, GB@vPLs exhibited a significantly accelerated decline in Cy5.5 signal intensity in plasma post‐injection, manifesting in a plasma half‐life of approximately 0.90 h for GB@vPLs, whereas GB@vPCs extended this period to about 3.23 h. This disparity could be attributed to the less stable liposomal components, which were prone to rapid clearance; conversely, the cerasome‐based GB@vPCs maintain a relatively stable state in plasma, thereby achieving an extended circulation time, crucial for the subsequent accumulation at tumor sites.

Subsequently, near‐infrared fluorescence imaging validated GB@vPCs' delivery and accumulation efficacy in tumors. In this experiment, mice were administered with DSPE‐Cy5.5‐labeled GB@vPCs, GB@vPLs, and GB@PCs, and the Cy5.5 fluorescence signals were tracked in real time. As shown in Figure 6b,d, by 1 h post‐injection, fluorescent signals were detected in the tumor regions of all three groups, with intensities gradually increasing over time and peaking at 24 h, following the gradient: GB@vPCs > GB@PCs > GB@vPLs. Notably, during the subsequent 120 h, GB@vPCs, and GB@PCs maintained stable fluorescence intensities in the tumor areas, whereas GB@vPLs showed a declining trend, highlighting cerasomes' superior retention capacity in the tumor region compared to liposomes. Furthermore, the var7 peptide modification on the cerasomes was demonstrated to enhance their selective accumulation at the tumor site. This finding is consistent with our previous study, neutralization of the acidic tumor microenvironment via oral and intratumoral bicarbonate administration markedly reduced tumor targeting, confirming that Var7 actively targets acidic tumors [52]. To further corroborate these findings, major organs from mice in each group were harvested at the end of the experiment for ex vivo fluorescence imaging. Figure 6c,e illustrated that the tumor regions of GB@vPCs‐treated mice displayed significantly brighter fluorescence compared to control groups, aligning closely with the in vivo dynamic observations. This conclusively demonstrates GB@vPCs' exceptional tumor‐targeting capability and prolonged retention, providing highly favorable conditions for implementing subsequent ultrasound‐triggered immunotherapy regimens.

Following this, we proceeded to investigate the performance of GB@vPCs as ultrasound contrast agents. Initially, we assessed their ultrasound contrast capabilities in vitro. As shown in Figure 6f, compared to the PBS control group, GB@vPCs demonstrated a significant enhancement in ultrasound contrast under ultrasound imaging (MI 0.09/ TIS 0.1, AP 50%, 3 MHz), with this enhancing effect showing a concentration‐dependent pattern. Upon application of ultrasound irradiation (1 MHz, 1 W cm^−2^, 20% duty cycle), a conspicuous reduction in the ultrasound signal was observed, suggesting that the signal attenuation leading to disappearance might result from alterations in particle size (Figure 6g). Next, we conducted similar tests on GB@vPCs loaded with oxygen (GB@vPCs (O_2_)). As shown in Figure 6g, akin to GB@vPCs without oxygen loading, GB@vPCs (O_2_) also displayed ultrasound contrast functionality under non‐ultrasound conditions. However, distinctively, when subjected to ultrasound (1 MHz, 1 W/cm^2^, 20% duty cycle), GB@vPCs (O_2_) retained a pronounced contrast ability. The oxygen contained within was released, and the resulting microbubbles of oxygen significantly amplified the ultrasound backscatter signal, a feature that holds great significance for real‐time monitoring of the oxygen release process. To further validate its in vivo applicability, we first examined GB@vPCs(O_2_)'s imaging performance in tumor sites via intratumoral injection, as shown in Figure S15a. Following the intratumoral administration of GB@vPCs (O_2_), a cluster‐like signal enhancement was observed in the tumor area post US. Subsequently, GB@vPCs (O_2_) were intravenously injected into 4T1 tumor‐bearing mice, and the tumor region was monitored using an ultrasound imaging system (Figure 6h). It was observed that within the initial 12 h post‐injection, no prominent ultrasound signal was detected in the tumor region, possibly due to the initially low concentration of nanodroplets in the tumor area. However, 24 h post‐IV injection, ultrasonic signals began to emerge within the tumor, further intensifying at the 48‐h mark, indicating the gradual accumulation of GB@vPCs(O_2_) in the tumor site. When ultrasound was applied to the tumor at the 48‐h point, a marked increase in the ultrasound signal was observed, reflecting oxygen release.

Additionally, we employed photoacoustic imaging to assess GB@vPCs(O_2_)'s efficacy in alleviating tumor hypoxia. As shown in Figure 6i, before drug administration, the tumor interior displayed a large distribution of blue color, indicative of severe hypoxia. 24 h post‐injection, parts of the previously blue vasculature within the tumor started turning red, and by 48 h, red signals emerged within the tumor, clearly demonstrating the progressive release of oxygen carried by GB@vPCs over time. Critically, after applying ultrasound at 48 h, the red signal was further augmented, with the majority of the tumor tissue transitioning to red. In contrast, the control group (with PBS injection) showed no significant change upon ultrasound exposure (Figure S15b), powerfully confirming that ultrasound triggers rapid oxygen release from encapsulated GB@vPCs, effectively ameliorating the hypoxic environment within the tumor.

### 2.8 Optimizing the Timing of the Sequencing of Chemotherapy and Immunotherapy

**FIGURE 7:**
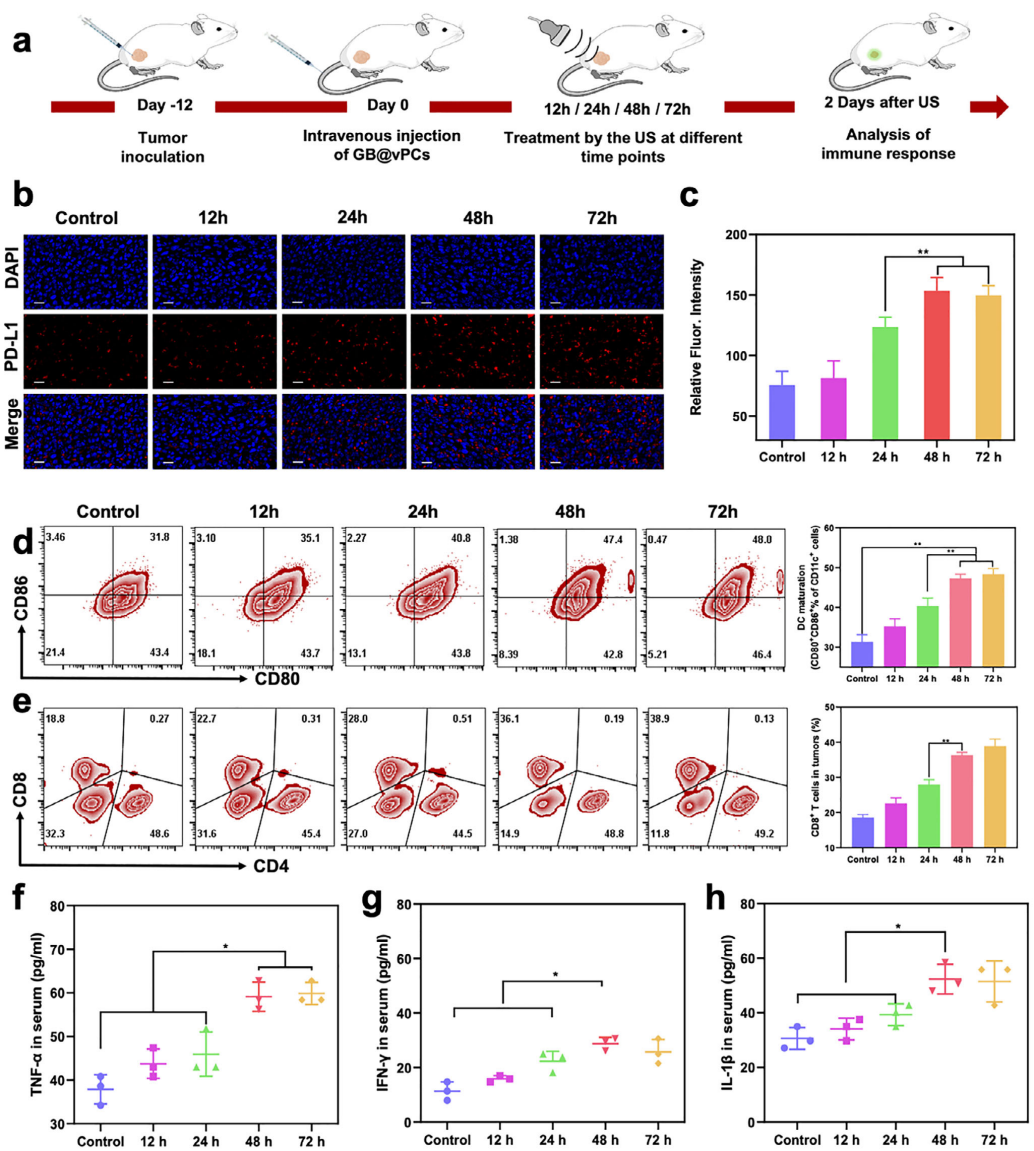
Optimization of the timing for the combined application of chemotherapy and immunotherapy. (a) Schematic illustration of the treatment regimen in mice; (b) representative immunofluorescence images depicting PD‐L1 expression on tumor cells at different time points after GEM treatment; (c) quantification of the PD‐L1 fluorescence intensity from (b); (d) flow cytometry analysis showing the percentage of mature DCs in tumor‐draining lymph nodes and the corresponding quantitative analysis; (e) flow cytometry analysis depicting the percentages of CD4^+^ and CD8^+^ lymphocytes in tumors and the corresponding quantitative analysis; (f–h) ELISA detection of (f) TNF‐α, (g) IFN‐γ, and (h) IL‐1β levels in mouse serum.

We initially investigated the impact of chemotherapeutic agents on PD‐L1 expression on the surface of tumor cells at various time points in vivo. A 4T1 tumor‐bearing mouse model was established, and upon tumors reaching ∼100 mm^3^, G@vPCs were administered via intravenous injection. Tumor samples were collected at 12, 24, 48, and 72 h post‐injection, followed by immunofluorescence staining and analysis (Figure 7a). Results depicted in Figure 7b,c reveal a marked increase in PD‐L1 expression on tumor cells over time, peaking at 48 h, emphasizing the time‐dependent effect of GEM chemotherapy on upregulating PD‐L1 in tumor cells.

To determine the optimal timing for ultrasound‐induced BMS1166 release, 4T1 tumor‐bearing mice with tumors reaching ∼100 mm^3^ were intravenously injected with GB@vPCs, and ultrasound was applied at varying intervals post‐injection. Two days after ultrasound, DC maturation in tumor‐draining lymph nodes was analyzed. DC maturation increased progressively from 31.4% (control) to 35.1%, 40.8%, 47.4%, and 48% at 12, 24, 48, and 72 h post‐injection, respectively (Figure 7d). No significant difference was observed between the 48 and 72 h time points (p > 0.05), indicating a plateau at ≥48 h. Both 48 and 72 h groups showed significantly higher DC maturation than all earlier time points (*p* = 0.001 vs. 24 h; *p* < 0.001 vs. control).

Notably, DC maturation was significantly enhanced following ultrasound exposure at ≥48 h, potentially due to peak immune stimulation by GEM chemotherapy coinciding with optimal BMS1166‐induced DC maturation and tumor accumulation of GB@vPCs. It should be noted the identified therapeutic window (≥48 h) may not solely reflect the intrinsic kinetics of PD‐L1 regulation, but rather the coordinated effects of nanoparticle tumor accumulation, sequential drug activation, and immune response dynamics. As demonstrated by the in vivo fluorescence imaging results, GB@vPCs progressively accumulated within the tumor after systemic administration, which may influence the efficacy of ultrasound‐triggered BMS1166 activation and subsequent immune modulation. Therefore, the favorable therapeutic efficacy observed at ≥48 h should be interpreted as an experimentally determined therapeutic window under the current conditions, rather than a universally optimal time point. Future studies aimed at independently regulating tumor accumulation and drug activation kinetics will further clarify the individual contributions of these processes.

Investigation into CD8+ T cell infiltration in tumors (Figure 7e) revealed that while the control group had a mere 18.8% CD8+ T cells, delaying ultrasound exposure led to a gradual increase, reaching 38.9% at 48 h and remaining at a comparable level at 72 h, indicating an optimal interval of ≥48 h. This reinforces that immune stimulation reaches a maximal level at ≥48 h post‐chemotherapy injection, with concurrent immunotherapy eliciting the most robust immune response.

Furthermore, key cytokines in the serum – interferon‐gamma (IFN‐γ), tumor necrosis factor‐alpha (TNF‐α), and interleukin‐1 beta (IL‐1β) – displayed congruent trends (Figure 7f–h). IFN‐γ and TNF‐α, indicators of T cell activity, and IL‐1β, associated with inflammatory necrosis, experienced changes consistent with the notion that chemotherapy and immunotherapy, when combined ≥48 h post‐injection, elicit the most potent immune and inflammatory responses. Notably, the levels of these cytokines, together with CD8^+^ T cell infiltration and DC maturation, plateaued at approximately 48 h; therefore, the 48 h time point was selected for ultrasound treatment in all subsequent experiments for practical convenience.

### 2.9 In Vivo Evaluation of the Anti‐Tumor Efficacy and Immune Response Induced by GB@vPCs

**FIGURE 8:**
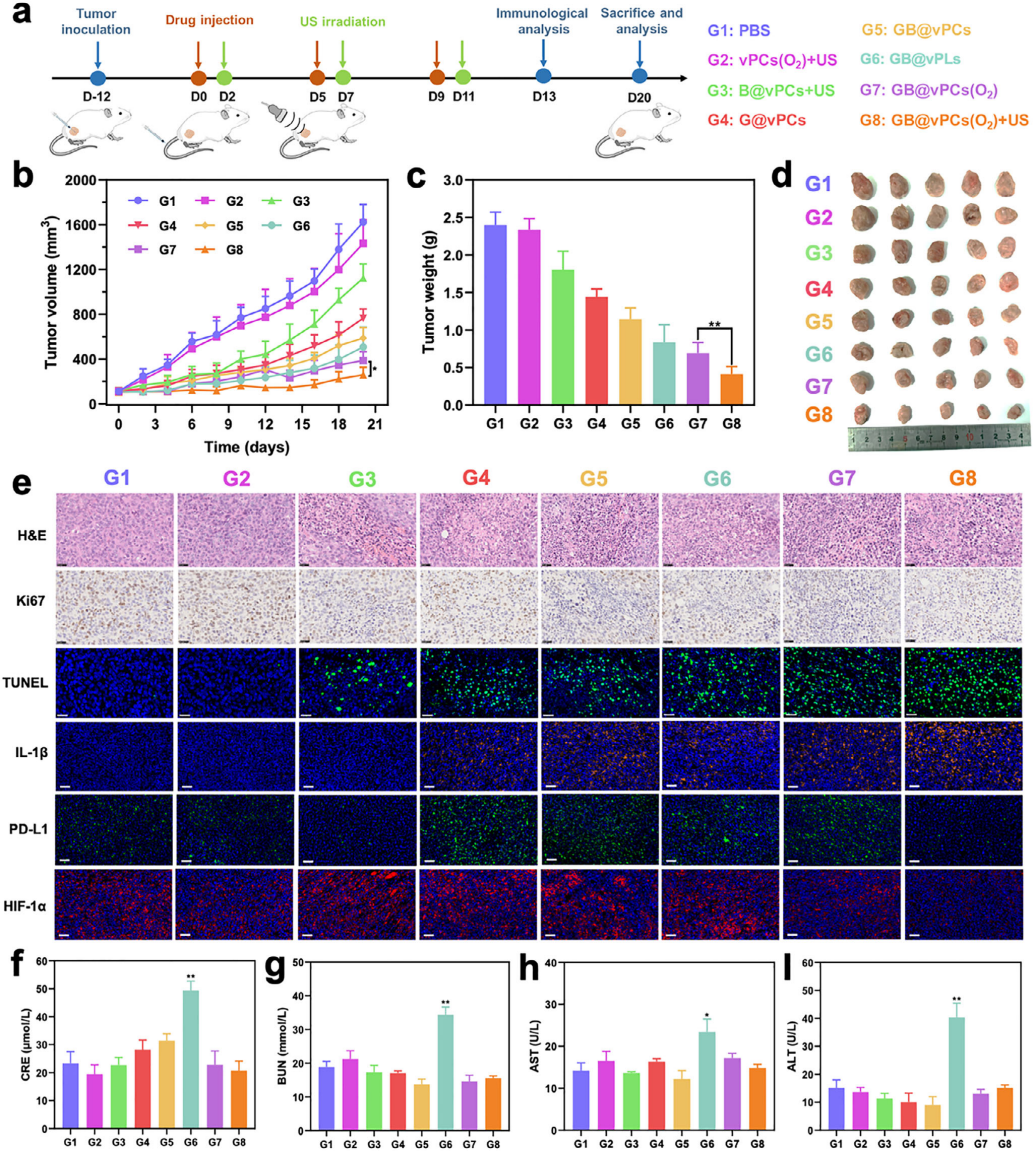
Anti‐tumor effects of GB@vPCs. (a) Schematic representation of the in vivo treatment protocol in mice; (b) tumor volume change over time in mice after various treatments; (c) tumor weight of mice in each group on day 20; (d) photographs of tumors taken on day 20 of treatment; (e) H&E staining, Ki67, TUNEL, IL‐1β, PD‐L1, and HIF‐1α assay results from tumors on day 13 of treatment (Scale bars: 25 µm for H&E and Ki67, 50 µm for the rest); (f) serum creatinine (CRE) levels, (g) Serum urea nitrogen (BUN) levels, (h) aspartate aminotransferase (AST) levels, and (i) alanine aminotransferase (ALT) levels measured from blood samples collected on day 20 of treatment.

To assess in vivo therapeutic efficacy, 4T1 tumor‐bearing BALB/c mice were randomly allocated into eight groups (n = 5): PBS, vPCs(O_2_) + US, B@vPCs + US, G@vPCs, GB@vPCs, GB@vPLs, GB@vPCs(O_2_), and GB@vPCs(O_2_) + US. Treatments were administered intravenously on days 0, 5, and 9, with ultrasound applied 48 h post‐injection (Figure 8a). By day 20, PBS and vPCs(O_2_) + US groups showed rapid tumor growth (∼1620 and ∼1430 mm^3^; *p* = 0.074), confirming carrier and ultrasound safety. B@vPCs + US moderately inhibited tumor growth (∼1100 mm^3^; *p* < 0.05 vs. PBS). G@vPCs and GB@vPCs further reduced tumor volumes to ∼760 and ∼580 mm^3^ (*p* < 0.001 vs. PBS), with GB@vPCs significantly lower than G@vPCs. GB@vPLs showed comparable efficacy to GB@vPCs (∼510 mm^3^; *p* = 0.122). GB@vPCs(O_2_) achieved greater suppression (∼380 mm^3^; *p* < 0.001 vs. GB@vPCs). GB@vPCs(O_2_) + US exhibited the strongest tumor inhibition (∼260 mm^3^; *p* = 0.0043 vs. GB@vPCs(O_2_); *p* < 0.001 vs. PBS) (Figure 8b). Tumor weights confirmed these trends (Figure 8c), and representative excised tumors are shown in Figure 8d. Body weights remained stable across all groups (Figure S16). Liver and kidney function markers of AST, CRE, ALT and BUN were significantly elevated only in the GB@vPLs group (*p* < 0.001, *p* = 0.0259/ 0.038/ 0.039 vs. PBS), likely attributable to premature BMS1166 leakage from the unstable liposomal carrier, whereas all other groups showed no abnormalities (*p* > 0.05) (Figure 8f–i).

Histopathological analysis was performed to evaluate treatment‐induced tumor cell apoptosis, proliferation, and hypoxia relief (Figure 8e). H&E staining revealed intact nuclei in PBS and vPCs(O_2_) + US groups, whereas all other treatment groups exhibited progressive nuclear shrinkage and fragmentation, with GB@vPCs(O_2_) + US showing the most extensive damage. TUNEL staining confirmed the highest level of apoptosis in the GB@vPCs(O_2_) + US group, and Ki67 staining demonstrated the lowest proliferation. Elevated IL‐1β expression in GEM‐treated groups indicated GEM‐induced pyroptosis. PD‐L1 immunofluorescence showed markedly increased PD‐L1 expression in G@vPCs, GB@vPCs, and GB@vPCs(O_2_) groups, consistent with GEM‐mediated PD‐L1 upregulation, whereas B@vPCs + US and GB@vPCs(O_2_) + US exhibited substantially reduced PD‐L1 levels due to ultrasound‐triggered BMS1166 release. HIF‐1α staining revealed severe hypoxia in PBS, B@vPCs + US, G@vPCs, GB@vPCs, and GB@vPLs groups, while vPCs(O_2_) + US, GB@vPCs(O_2_), and GB@vPCs(O_2_) + US showed markedly reduced HIF‐1α expression, with GB@vPCs(O_2_) + US exhibiting the strongest hypoxia relief, attributable to the combined effects of oxygen supply and superior tumor growth inhibition.

**FIGURE 9:**
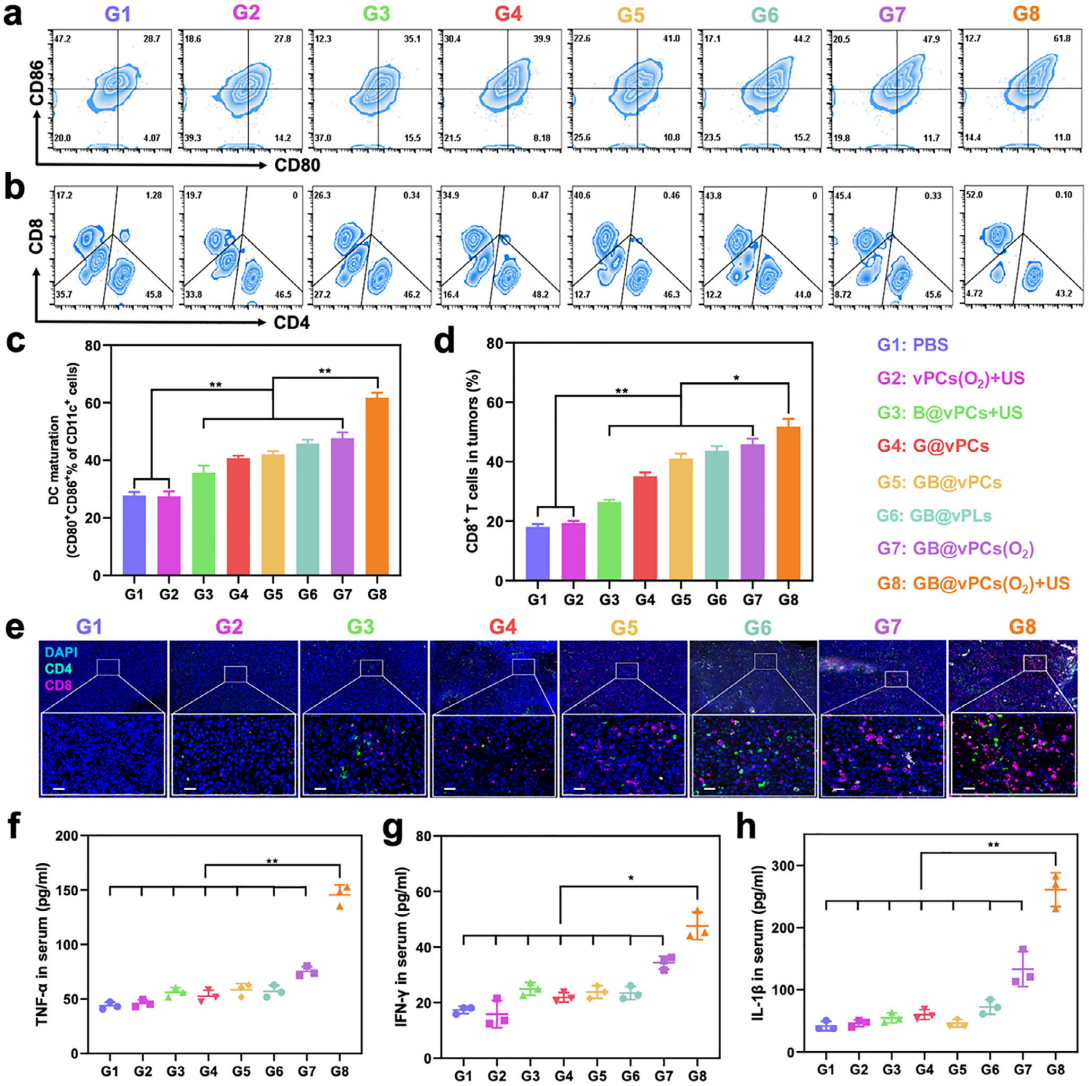
Immune response mediated by GB@vPCs. (a) Flow cytometry analysis of the percentage of mature DCs in tumor‐draining lymph nodes on day 13 of treatment; (b) flow cytometry analysis of the percentage of CD4^+^ and CD8^+^ lymphocytes in tumors on day 13 of treatment; (c) quantitative analysis corresponding to (a); (d) quantitative analysis corresponding to (b); (e) immunofluorescence images of CD4^+^ and CD8^+^ infiltrating lymphocytes within tumors on day 13 of treatment (scale bar = 25 µm); ELISA detection of (f) TNF‐α, (g) IFN‐γ and (h) IL‐1β levels in mouse serum on day 13 of treatment.

Following three therapeutic cycles, immune responses in tumor‐draining lymph nodes and tumors were evaluated by flow cytometry. As shown in Figure 9a,c, DC maturation was comparable between PBS (∼28.7%) and B@vPCs + US (∼27.8%) groups (*p* > 0.05). In contrast, G@vPCs, GB@vPCs, and GB@vPCs(O_2_) significantly increased DC maturation (*p* < 0.001 vs. PBS), attributed to GEM‐induced ICD and pyroptosis. GB@vPLs also enhanced DC maturation (*p* < 0.001 vs. PBS), reflecting the combined effect of GEM and BMS1166. Notably, GB@vPCs(O_2_) + US achieved the highest DC maturation (∼61.8%), significantly greater than all other groups (*p* < 0.001 vs. GB@vPCs(O_2_)), demonstrating the synergistic effect of chemotherapy, hypoxia relief, and ultrasound‐triggered BMS1166 release.

Flow cytometry analysis of intratumoral CD8^+^ T cell infiltration (Figure 9b,d) revealed that CD8^+^ T‐cell infiltration increased in the drug‐containing treatment groups, with GB@vPCs(O_2_) + US exhibiting the highest infiltration (*p* = 0.005 vs. GB@vPCs(O_2_) and *p* < 0.001 vs. PBS). Immunofluorescence staining of tumor tissues confirmed these results, showing the strongest CD8^+^ signal in the GB@vPCs(O_2_) + US group (Figure 9e). Consistently, serum levels of IFN‐γ, TNF‐α, and IL‐1β were highest in the GB@vPCs(O_2_) + US group (Figure 9f–h), significantly exceeding those in all other groups (*p* = 0.0015/ P< 0.001/ *p*< 0.001 vs. GB@vPCs(O_2_)). Collectively, GB@vPCs(O_2_) combined with ultrasound not only exerts a pronounced antitumor effect but also potently activates T‐cell‐mediated immune responses, achieving specific immunotherapy.

Nevertheless, while sequential release is an important feature, the superior therapeutic efficacy is likely attributable to the combined effect of the inherent synergy between chemotherapy and immunotherapy, rather than to sequential release alone. In addition, studies incorporating HIF‐1α gain‐of‐function experiments will be valuable to definitively clarify the contribution of hypoxia relief to the observed immune activation.

### 2.10 In Vivo Investigation of GB@vPCs' Ability to Suppress Distant Tumors and Inhibit Metastatic Tumors

**FIGURE 10:**
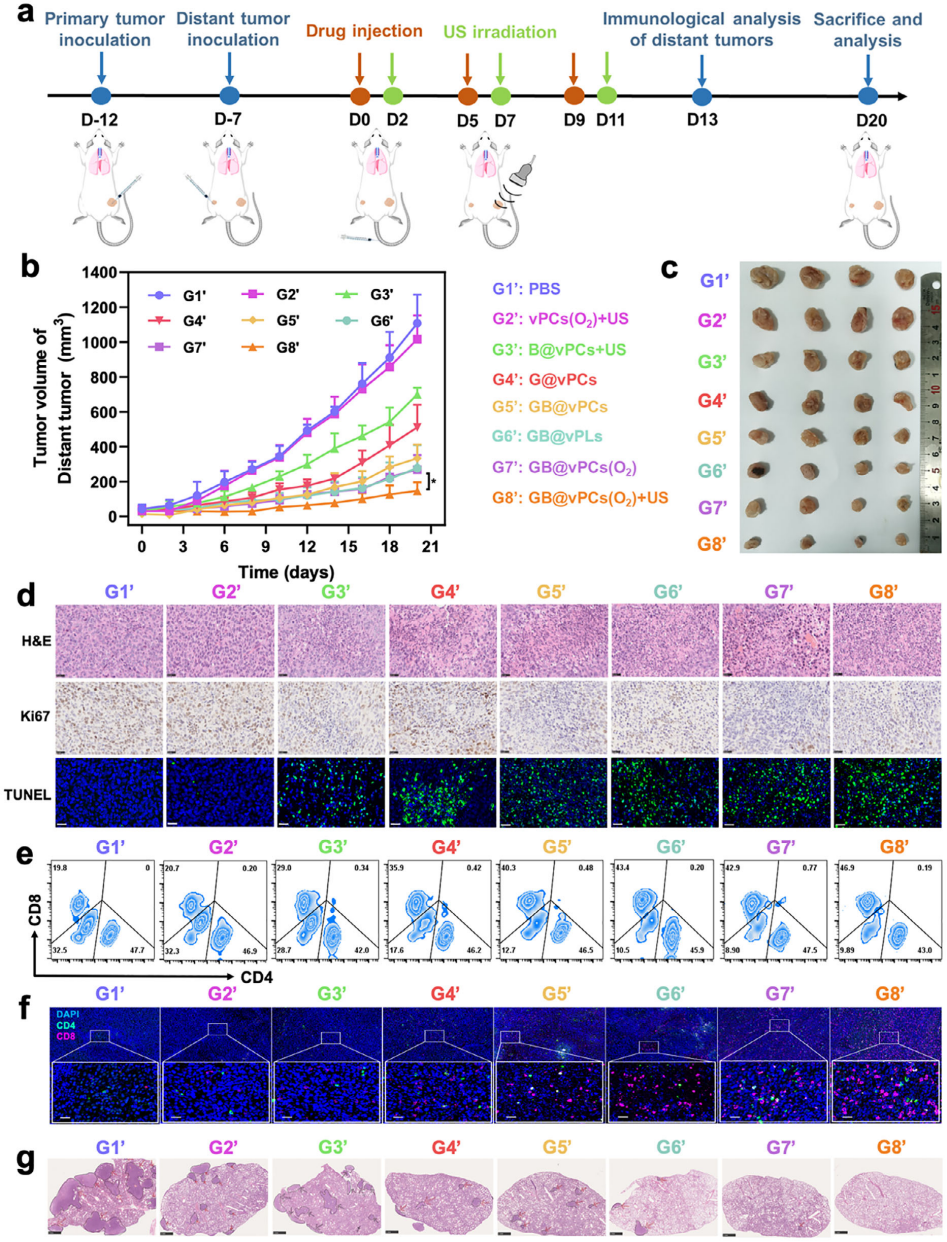
Ability of GB@vPCs to suppress distant tumors. (a) Schematic representation of the treatment protocol for distant tumors in mice; (b) distant tumor volume change over time in mice after various treatments; (c) photographs of distant tumors taken on day 20 of treatment; (d) H&E staining, Ki67, and TUNEL assay results from distant tumors on day 13 of treatment (scale bars: 25 µm for H&E and Ki67, 50 µm for TUNEL); (e) flow cytometry analysis of the percentage of CD4^+^ and CD8^+^ lymphocytes in distant tumors on day 13 of treatment; (f) immunofluorescence images of CD4^+^ and CD8^+^ infiltrating lymphocytes within distant tumors on day 13 of treatment (scale bar = 25 µm); (g) H&E staining of mouse lungs on day 20 of treatment (scale bar = 1 mm).

Given that GB@vPCs(O_2_) combined with US efficiently inhibited primary tumors, we further studied its suppression on distant tumors via a bilateral‑tumor model. Mice bearing primary tumors on the right flank were inoculated with secondary tumors on the left flank five days later and randomized into eight groups (n = 4). Treatments were administered intravenously on days 0, 5, and 9, with ultrasound applied to the primary tumor 48 h post‐injection (Figure 10a). Distant tumors in PBS and vPCs(O_2_) + US groups grew rapidly, with no significant difference between them (p = 0.414). B@vPCs + US, G@vPCs, GB@vPCs, GB@vPLs, and GB@vPCs(O_2_) significantly inhibited distant tumor growth compared with PBS. Notably, GB@vPCs(O_2_) + US achieved the strongest inhibition, with a trend toward greater suppression than GB@vPCs(O_2_), and significantly greater than all other groups (*p* < 0.001 vs. PBS, Figure 10b,c), indicating a potent systemic anti‐tumor immune response.

Histological examinations, including H&E staining, Ki67 immunohistochemistry, and TUNEL (Figure 10d), revealed the most severe pathology, least proliferation, and most apoptosis in the distant tumors of the GB@vPCs(O_2_) + US group. Additionally, flow cytometry and immunofluorescence analyses (Figure 10e,f) quantified CD8^+^ T cell infiltration in distant tumors, with GB@vPCs(O_2_) + US showing the highest infiltration, indicative of strong tumor suppression. At the study's end, HE staining of lungs were conducted (Figure 10g), revealing numerous metastatic nodules in the lungs of the PBS group. The vPCs(O_2_) + US group had fewer nodules, potentially due to the mitigating effect of oxygen supply on tumor invasiveness. Similarly, all treated groups except GB@vPCs(O_2_) + US showed reduced lung nodules, as chemotherapy and BMS1166‐induced immunotherapy enhanced T cell infiltration and immune responses. Remarkably, GB@vPCs(O_2_) + US mice had virtually no detectable nodules, indicating the superior antimetastatic effect of the combined treatment. Although GB@vPLs led to liver and kidney abnormalities, including vacuolation and inflammatory infiltration, pathologic examination of other organs beyond the lungs did not show overt damage. No significant organ pathology was observed in other treatment groups, further confirming treatment safety (Figure S17).

**FIGURE 11:**
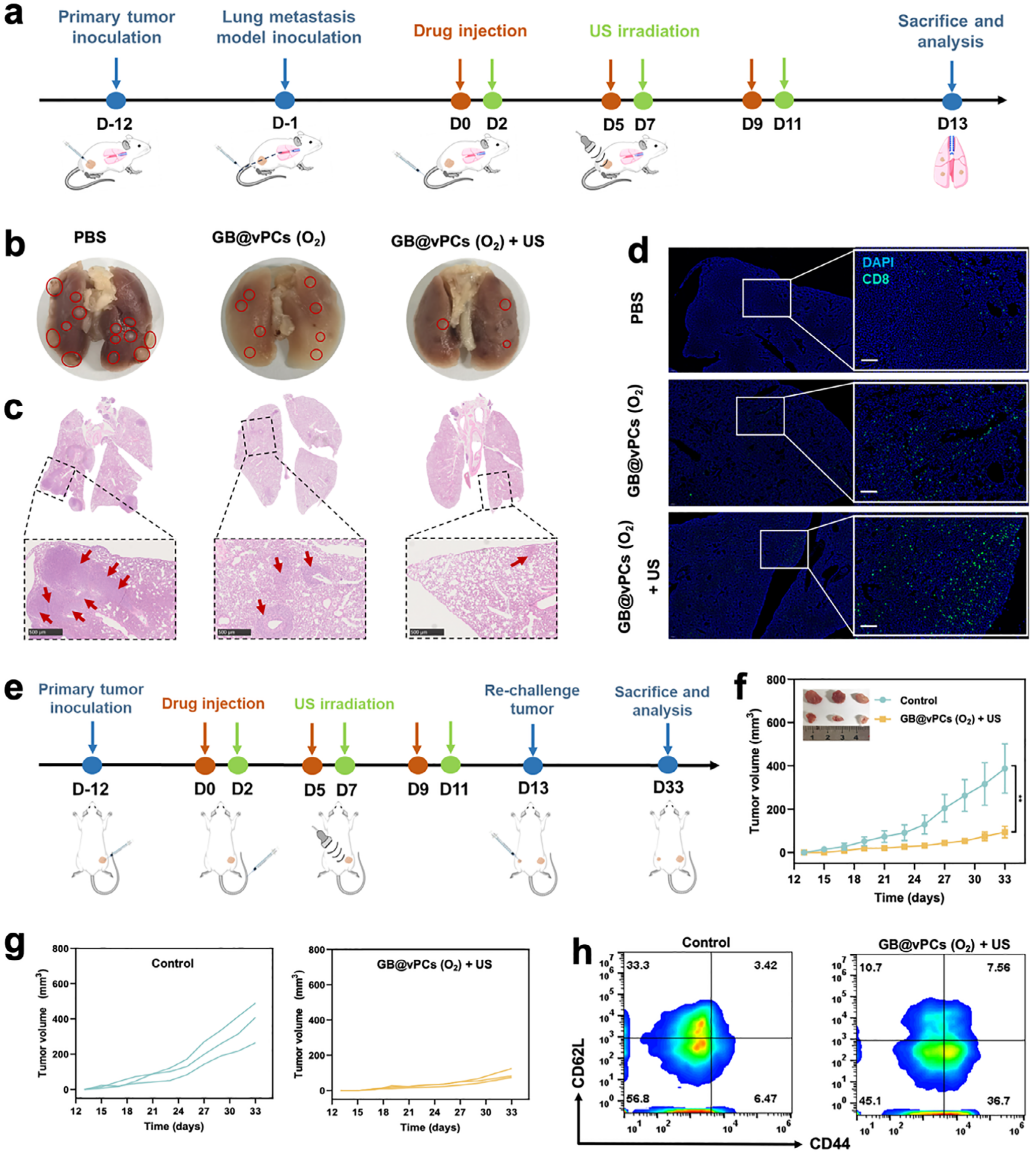
Suppression of lung metastasis by GB@vPCs. (a) Schematic illustration of the construction of a lung metastasis mouse model and the treatment regimen; (b) photographs of mouse lung tissues post‐treatment; (c) H&E staining of mouse lung sections post‐treatment (scale bar = 500 µm); (d) immunofluorescence images of CD8^+^ cells in mouse lung tissues post‐treatment (scale bar = 100 µm). (e) schematic illustration of the tumor rechallenge model and therapeutic schedule; (f) growth curves of secondary tumors over time (inset: photograph of excised tumors at endpoint); (g) individual secondary‐tumor growth curves for mice in each group; (h) flow cytometric analysis of splenic memory T cells.

To further investigate the capability of GB@vPCs(O_2_)+US therapy to inhibit pulmonary metastasis, we established a mouse model of pulmonary metastasis. The experimental protocol proceeded as follows: after establishing primary tumors in mice, 4T1 tumor cells were injected through the tail vein one day before drug administration to simulate lung metastasis. Thereafter, the primary tumors received the same three‐course treatment as before. Upon treatment completion, the development of pulmonary metastases was examined (Figure 11a). As shown in Figure 11b,c, the PBS group had noticeable metastatic foci in the lungs, while GB@vPCs(O_2_) mice showed a marked reduction in lung nodules, primarily due to chemotherapy‐induced immunogenic cell death (ICD) and pyroptosis processes that stimulate an immune response, thereby reducing lung metastases. Significantly, mice receiving GB@vPCs(O_2_)+US therapy had a further drastic decrease in lung nodules, attributed to the combined treatment strategy more effectively mobilizing the immune system and enhancing the clearance of pulmonary metastatic sites. Lung immunofluorescence staining (Figure 11d) revealed minimal CD8^+^ T cell infiltration in the metastatic areas of the lungs in the PBS group, whereas GB@vPCs(O_2_) and GB@vPCs(O_2_)+US treated mice showed extensive CD8^+^ T cell infiltration, with GB@vPCs(O_2_)+US showing the most remarkable infiltration, highlighting the combined treatment's superiority in boosting immune cell targeting and destruction of metastatic foci. Future studies incorporating the analysis of T cell exhaustion markers (e.g., PD‐1, TIM3) will be valuable to determine whether the anti‐metastatic effects are associated with the reversal of T cell exhaustion.

To explore the contralateral challenge‑associated protection triggered by GB@vPCs(O_2_) combined with US, we constructed a tumor rechallenge model (Figure 11e). Mice that responded to GB@vPCs(O_2_) + US therapy received contralateral 4T1 cell inoculation, and healthy mice were set as the control group. As presented in Figure 11f,g, the treatment group delayed tumor growth after rechallenge when compared with rapidly‑growing tumors in controls (*p* < 0.001). Flow‑cytometric detection of splenic effector‑memory T cells (TEMs) showed an elevated proportion in the GB@vPCs(O_2_) + US group (around 36.7%) relative to controls (around 6%) (*p* < 0.001, Figure 11h). These data suggest that GB@vPCs(O_2_) + US elicits immune‑related protection to slow tumor regrowth upon secondary tumor‑cell challenge. Future studies will incorporate the profiling of tumor‐resident memory CD8^+^ T cells (TRMs) and memory B cells to fully elucidate the tissue‐distribution mechanisms underlying the durable anti‐tumor immune memory observed in our models.

Several limitations of this study should be acknowledged. Firstly, this study was designed to evaluate the in vivo performance of co‐delivery systems that combine chemotherapeutic drugs with immune adjuvants. A direct in vivo comparison of free gemcitabine and free BMS1166 is not straightforward, as it is not possible to unify tumor enrichment and pharmacokinetic profiles between these two drugs. Future studies using alternative comparator designs, such as free drugs at maximum tolerated dose or clinically relevant dosing schedules, will be needed to establish the translational advantage of the carrier‐mediated approach. Besides, Real‐time kinetic monitoring of drug release will be necessary to achieve true precision control. Thirdly, future studies should prioritize comprehensive survival analysis with extended observation periods to fully evaluate the long‐term durability of the therapeutic responses. Subsequent work will focus on the clinical translation of this formulation, including the development of lyophilization protocols and excipient screening to further extend the storage duration.

## 3 Conclusion

In this study, a dual‐stimuli‐responsive and highly stable targeted silicate‐based cerasome drug delivery system was constructed to achieve spatiotemporal controlled release of the chemotherapeutic drug GEM and the immune checkpoint inhibitor BMS1166 within the tumor microenvironment, thereby regulating the different release timepoints of different drugs at the same action site to induce robust immunotherapy efficacy. In this approach, GEM is covalently bound to the silicate surface via a disulfide linkage, enabling selective release in the high‐glutathione environment of tumors to initially deliver cytotoxic chemotherapy and stimulate local immune responses. Subsequently, at an appropriate time point, ultrasound activation triggers increased permeability of the silicate vesicle membrane, ensuring the subsequent release of the encapsulated BMS1166, effectively blocking the PD‐1/PD‐L1 signaling pathway and breaking the tumor's immune tolerance. To enhance the precision of drug delivery, the acid‐targeting peptide lipid Var7 was introduced, enabling the silicate carrier to accurately target and concentrate within the unique acidic microenvironment of tumor tissues, ensuring the efficient accumulation of drugs in the diseased area. This system not only significantly inhibits the growth of primary tumors but also demonstrates a powerful capacity to suppress distant tumors and lung metastasis.

## 4 Materials and Methods

### 4.1 Materials

The N‐[N‐(3‐triethoxysilylpropyl)aminopropylcarbonylamino] succinamide, referred to as the silica‐forming lipid (CFL), was synthesized following previously reported methods [62]. 1,2‐Distearoyl‐sn‐glycero‐3‐phosphocholine (DSPC) and 1,2‐distearoyl‐sn‐glycero‐3‐phosphoethanolamine‐N‐[methoxy(polyethylene glycol)‐2000] (DSPE‐PEG2000) were purchased from AVT (Shanghai) Biotechnology Co., Ltd. 1,2‐distearoyl‐sn‐glycero‐3‐phosphoethanolamine‐N‐[methoxy(polyethylene glycol)‐2000]‐maleimide (DSPE‐PEG2000‐Maleimide) and Var7 peptide (sequence ACEEQNPWARYLEWLFPTETLLLEL) were obtained from Xi'an RuiXi Biological Technology Co., Ltd. CCK‐8 was obtained from Elabscience Co., Ltd. Annexin V‐FITC/PI apoptosis detection kit was purchased from KeyGen Biotech Co., Ltd. ELISA kits for murine IFN‐γ, TNF‐α, and IL‐1β were purchased from Solarbio Science & Technology Co., Ltd (SEKM‐0002‐48T, SEKM‐0031‐48T, SEKM‐0034‐48T). Fluorochrome‐conjugated antibodies were purchased from BioLegend. Antibodies against NLRP3, cleaved caspase‐1, GSDMD, and N‐GSDMD were purchased from Abcam, and anti‐P‐gp antibody was obtained from Biodragon (BD‐PT3692). Chemicals for high‐performance liquid chromatography (HPLC) were of chromatographic grade, while all other chemicals were analytical grade.

### 4.2 Synthesis of GEM‐Lip

The synthesis of GEM‐Lip involves two critical steps. First, the intermediate 2 was synthesized (Figure S1). The procedure was as follows: Accurately weighed 2 grams of Compound **1** into a clean round‐bottom flask, then added 50 mL of DMF (dimethylformamide). Sequentially, introduced 2.35 mL of 2,2'‐ dithiodiethanol into this solution slowly. Following this, according to a specific molar ratio—Compound **1**: 2,2'‐ Dithiodiethanol: DMAP: EDC at 1:5:2:0.4—add DMAP and EDC catalyst sequentially to ensure smooth esterification. The assembled reaction system was placed on a magnetic stirrer and stirred overnight at 50°C under a nitrogen atmosphere to ensure a complete reaction. Post‐reaction, DMF solvents were removed using a rotary evaporator, and the product was purified via silica gel chromatography, yielding the target intermediate 2. Characterization and identification of the Compound **2** were carried out using mass spectrometry.

In the second step of synthesizing GEM‐Lip, 1.7 grams of intermediate 2 were dissolved with DIPEA (N,N‐diisopropylethylamine) and pyridine in dichloromethane according to a molar ratio of 1: 4: 0.5. The reaction mixture was placed in a 0°C water bath, and then, 4‐Nitrophenyl chloroformate was added dropwise in a molar ratio of Compound **2**: 4‐Nitrophenyl chloroformate at 1: 3 under room temperature and stirred for 4 h. Subsequently, dichloromethane was evaporated by rotary evaporation, and DMF was added to the remaining solid. The required amount of GEM was introduced to Compound **2** at a molar ratio of 3: 1, along with TEA (triethylamine) as a reaction auxiliary, and stirred for another 4 h at room temperature. Afterward, solvents were removed by rotary evaporation, the reaction mixture was dissolved in dichloromethane and then purified by silica gel chromatography to obtain the target product GEM‐Lip, which was characterized using mass spectrometry.

### 4.3 Synthesis of DSPE‐PEG‐Var7

The synthesis of DSPE‐PEG‐Var7 was achieved through the conjugation of the thiol group of Var7 peptide with maleimide. In brief, DSPE‐PEG‐Mal and Var 7 peptide were mixed at a molar ratio of 1: 1.5 in anhydrous methanol. This mixture was then introduced into an aqueous phase composed of a volume ratio of 1 part anhydrous methanol to 9 parts doubly distilled water (ddH_2_O), followed by stirring at room temperature for 24 h to yield DSPE‐PEG‐Var7. Upon completion of the reaction, the crude product was dispersed in water and subjected to dialysis (using a membrane with a molecular weight cut‐off, MWCO, ranging from 8000 to 14 000) for 24 h to remove any unbound Var7 peptide molecules. The product, after dialysis purification, was lyophilized to obtain a solid powder. The resulting compound was characterized using Fourier Transform Infrared (FTIR) spectroscopy (Nicolet iS10, Thermo Fisher Scientific) and Matrix‐Assisted Laser Desorption/Ionization Time‐of‐Flight Mass Spectrometry (MALDI TOF MS) (UltrafleXtreme, Bruker).

### 4.4 Preparation and Formulation Optimization of GB@vPCs

The preparation of GB@vPCs employed the ethanol injection method. Initially, three components–‐CFL, GEM‐Lip, and DSPE‐PEG2000—were dissolved in ethanol at a molar ratio of 55:30:10. Separately, DSPE‐PEG‐Var7, accounting for 5% of the total lipid molar amount, was dissolved in DMF. These two solutions were then combined. Subsequently, BMS1166 was dissolved in DMSO and added to the lipid solution at various drug‐to‐lipid mass ratios (1:20, 1:15, 1:10, 1:5, and 1:4) to determine the optimal loading conditions. This mixture was then rapidly dispersed under ultrasonic bath conditions into doubly distilled water (ddH_2_O), yielding a uniform milky suspension, which was then dialyzed against ddH_2_O for 1 h at room temperature using a dialysis bag with a molecular weight cut‐off (MWCO) of 8000–14 000 to remove organic solvents. Following this, 20 µL of PFH was added per milligram of lipid component, and probe sonication was applied to form GB@vPCs. Unencapsulated free BMS1166 was removed through purification using a Sephadex G‐50 dextran gel column. The GB@vPCs were allowed to stand at room temperature for 24 h to facilitate the condensation reaction of surface silanol groups, leading to the formation of a stable silicate network structure. For oxygen loading, GB@vPCs were placed in an oxygen‐saturated chamber for 60 min to ensure full oxygen saturation, ultimately yielding oxygen‐saturated GB@vPCs(O_2_) nanodroplets. The control Cerasomes were prepared similarly via ethanol injection. A lipid mixture of CFL and DSPE‐PEG2000 (90:10 molar ratio) in ethanol was rapidly injected into ddH2O under sonication. After dialysis to remove ethanol, the suspension was left standing at room temperature for 24 h to allow silanol condensation, forming the final stable Cerasome structure.

In the preparation of GB@vPCs, to ascertain the optimal ratio between GEM‐Lip and CFL, a series of combinations with different molar ratios of GEM‐Lip to CFL (specifically, 20:65, 30:55, 40:45, and 50:35) were designed, and corresponding GB@vPCs nanodroplets were fabricated following the described method. Simultaneously, as a control, GB@vPLs devoid of silicate were prepared by substituting DSPC for CFL. The selection of the appropriate drug carrier formula was based on the performance of BMS1166 release. Moreover, during the GB@vPCs fabrication, a range of GB@vPCs samples were prepared by mixing BMS1166 with various lipid components at different mass ratios, and subsequently, their encapsulation efficiency and drug loading content were determined to identify the optimal drug‐to‐lipid ratio. To accurately evaluate encapsulation efficiency and drug loading, the purified GB@vPCs were first incubated overnight in acidic ethanol (1 M HCl) under vigorous stirring to disrupt the cerasomal structure and liberate BMS1166. Thereafter, the released BMS1166 was quantitatively analyzed using high‐performance liquid chromatography (HPLC). The mobile phase comprised acetonitrile and ddH_2_O containing 0.05% trifluoroacetic acid, with a dynamic gradient program set as follows: starting at 0 min time with a volume ratio of acetonitrile: ddH_2_O = 10:90, adjusting to 90:10 at 8 min, switching to 100:0 at 8.5 min, maintaining 100:0 until 11.5 min, reverting to 10:90 at 12 min, and sustaining this ratio until 16 min. The flow rate throughout was set at 1 mL min^−1^, and BMS1166 was detected at a wavelength of 254 nm. The encapsulation efficiency and drug loading content were calculated using the following formulas:

$$\begin{array}{ccc} & & \mathrm{Drug}\;\mathrm{Encapsulation}\;\mathrm{Efficiency}\;\\ & & \;=\;(Mass\;of\;BMS1166\;in\;Nanodroplets\;/\\ & & \;\;Actual\;Input\;Mass\;of\;BMS1166)\times 100\% \end{array}$$

$$\begin{array}{ccc} & & \mathrm{Drug}\;\mathrm{Loading}\;\mathrm{Content}\\ & & \;=[Mass\;of\;BMS1166\;in\;Nanodroplets\;/\\ & & \;\;(Total\;Mass\;of\;Nanoparticles+Drug)]\times 100\% \end{array}$$

The release behavior of GEM from G@vPCs under different concentrations of DTT and pH conditions was evaluated using dialysis. Initially, 1 mL of the G@vPCs solution was loaded into a dialysis bag, which was then immersed in phosphate‐buffered saline (pH 7.4, containing 0.1% Tween‐80) with varying concentrations of DTT (0 mM, 1 mM, 5 mM, and 10 mM) in a thermostatic shaker maintained at 37°C and 120 rpm to monitor the GEM release process. At predetermined time points, 1 mL of the release medium was withdrawn and immediately replenished with an equal volume of fresh buffer to maintain a constant volume. The withdrawn release media were analyzed by HPLC to determine GEM content. HPLC parameters were set as follows: the mobile phase consisted of a 90:10 volume ratio of methanol to 0.05 M ammonium acetate buffer, with a flow rate of 1 mL min^−1^, and GEM was detected at a wavelength of 268 nm. When investigating the impact of different pH values on GEM release, the above procedures were repeated, but with the G@vPCs solution in the dialysis bag immersed in phosphate buffer of different pH values (5.0, 6.5, 7.4) without DTT, while all other experimental conditions remained consistent with the previous release experiments.

The release behavior of BMS1166 from GB@vPCs was assessed using a centrifugation method. A volume of 1 mL of GB@vPCs was placed in a thermostatic shaker, where the release process of BMS1166 was monitored under conditions of 37°C and 120 rpm. At pre‐set time intervals, samples were taken out and subjected to centrifugation at 2000 rpm. Owing to their larger mass and density, GB@vPCs sedimented to the bottom during centrifugation, whereas the released BMS1166, although hydrophobic, remained in the supernatant due to its smaller molecular weight. Following this, 200 µL of the supernatant was collected and dissolved in DMSO and subsequently quantified through HPLC analysis. In studying the ultrasound‐triggered release of BMS1166, the samples were placed in vials and subjected to ultrasound irradiation at the 1‐hour and 3‐hour time points. Ultrasound parameters were set to a frequency of 1.0 MHz, a power density of 1 W cm^−2^, and a duty cycle of 50%, with each ultrasound exposure lasting for 5 min.

The oxygen release performance was measured using a dissolved oxygen meter (model AZ8403, China). GB@vPCs, after being loaded with oxygen to form GB@vPCs(O_2_), underwent evaluation of their oxygen release properties. The specific procedure was as follows: 3 mL of nanodroplets at a concentration of 400 µg mL^−1^ were placed in a 10 mL vial. Initially, non‐encapsulated oxygen in the solution was displaced using nitrogen, following which the oxygen meter probe was submerged in the center of the solution and the vial was sealed. The influence of ultrasound irradiation on oxygen release was first examined, with various ultrasound parameters set: power densities of 1 W cm^−2^ (duty cycles of 20% and 50%) and 1.5 W cm^−2^ (duty cycles of 20% and 50%), with a unified frequency of 1 MHz. Oxygen concentrations in the solution were measured at predetermined time points with or without ultrasound irradiation. To investigate the impact of different nanodroplet concentrations on oxygen release, GB@vPCs(O_2_) at varying concentrations (200, 400, and 800 µg mL^−1^) were separately placed in vials, and a uniform ultrasound condition of 1 W cm^−2^ power density, 50% duty cycle, and 1 MHz frequency was applied. Oxygen concentrations in the solution at different time points were recorded and compared across the various concentrations.

### 4.5 Cell Culture Study

The 4T1 mouse breast cancer cell line was acquired from the American Type Culture Collection (ATCC) and was cultivated in Roswell Park Memorial Institute (RPMI) 1640 medium (manufactured by Gibco, USA), which was further enriched with 10% premium fetal bovine serum (also from Gibco, USA) and 1% penicillin‐streptomycin solution (Gibco, USA). The cells were grown in an incubator set at a stable temperature of 37°C, with a 5% carbon dioxide (CO_2_) level and optimal humidity, ensuring a conducive environment for cell proliferation.

### 4.6 In Vitro Cellular Uptake

The uptake of nanodroplets in 4T1 cells and their acidity‐sensitive tumor‐targeting capability were investigated both qualitatively and quantitatively using confocal laser scanning microscopy (CLSM, Nikon, Japan) and flow cytometry (Beckman Coulter Calibur2, USA). DSPE‐PEG‐FITC was used to fluorescently label GB@PCs and GB@vPCs, and the fluorescence intensity of FITC was detected to assess cellular uptake behavior. The procedure was as follows: Initially, 4T1 cells were seeded at a density of 1 × 10^5^ cells/well in confocal dishes and incubated overnight. Then, the old medium was replaced with a fresh medium containing GB@PCs and GB@vPCs (lipids, at 5 µg mL^−1^) and incubated for 2 h, 4 h at pH 7.2 and pH 6.0. After incubation, the medium was discarded, and cells were washed with PBS, followed by incubation with Lyso‐Tracker Red (from Invitrogen) for 30 min to stain lysosomes. Hoechst 33342 was used to stain cell nuclei for 10 min. CLSM was employed to image the stained cells. For flow cytometry analysis, cells were seeded at a density of 2 × 10^5^ cells/well in a 12‐well plate and incubated under the same conditions. Post‐incubation, cells were washed with PBS, harvested by centrifugation, resuspended in PBS, and analyzed using flow cytometry.

### 4.7 Immunophenotyping Assessment

To investigate the effect of the chemotherapeutic agent gemcitabine (GEM) on upregulating PD‐L1 expression on the surface of 4T1 cells, we first seeded 4T1 cells at a density of 2 × 10^5^ cells per well in a 12‐well plate and incubated them overnight. The original medium was then replaced with fresh medium containing free GEM at varying concentrations (0.005, 0.01, 0.02, and 0.04 µg mL^−1^) or G@vPCs nanodroplets conjugating GEM, and further incubated for 24 h. In another set of experiments, to observe the impact of incubation duration on PD‐L1 expression, with the GEM concentration fixed at 0.04 µg mL^−1^, cells were incubated for 24, 48, and 72 h. Post‐incubation, cells were washed with PBS, harvested by centrifugation, and subsequently stained with PE‐conjugated anti‐mouse PD‐L1 antibody (from Biolegend, USA) for 30 min to assess PD‐L1 expression. Cells were washed twice with PBS afterward to remove unbound antibodies. Finally, flow cytometry was employed to quantify the PE fluorescence signal on the cell membrane, thus analyzing changes in PD‐L1 expression levels on 4T1 cells induced by GEM at different concentrations and incubation times.

PD‐L1 blockade by BMS1166 was assessed by flow cytometry according to established protocols [63, 64, 65]. The experiment was divided into six groups: Negative Control, Positive Control, G@vPCs, BMS1166, GB@vPCs, and GB@vPCs + US. Except for the Negative Control group, all other groups were pretreated with IFN‐γ (100 ng mL^−1^) for over 24 h to modulate PD‐L1 expression on the cell membrane before drug incubation. Subsequently, corresponding drugs were added to the cells for a 4 h incubation. For the US group, following 4 h of drug‐cell incubation, ultrasound treatment (frequency 1.0 MHz, power density 1 W cm^−2^, duty cycle 20%, duration 5 min) was applied, followed by continued 12‐h incubation. After incubation, cells were washed with PBS, and all groups except the Negative Control were stained with PE‐labeled mouse PD‐L1 antibody for 30 min. After staining, cells were washed twice with PBS to remove unbound antibodies. Finally, flow cytometry was employed to detect the PE fluorescence signal on the cell membrane, thereby assessing BMS1166‐mediated inhibition of the PD‐1–PD‐L1 interaction in 4T1 cells.

To study the immunogenic death of 4T1 cells induced by chemotherapy, we first assessed the degree of calreticulin (CRT) exposure on the cell surface post‐treatment using confocal laser scanning microscopy (CLSM) and flow cytometry. First, 1 × 10^5^ cells per dish were seeded in a specialized dish for confocal imaging and incubated overnight. The medium was then replaced with fresh medium containing vPCs, BMS1166, Free GEM, G@vPCs, and GB@vPCs (with GEM concentration set at 0.04 µg mL^−1^, other drug components equivalent to the amounts in GB@vPCs) and incubated for 24 h. After incubation, cells were washed with PBS, blocked with goat serum for 1 h, and incubated with CRT primary antibody (ab92516, Abcam) for 1 h, and secondary antibody (ab150078, Abcam) for 30 min. Following two washes with PBS, cells were fixed and stained with DAPI before imaging by CLSM. For flow cytometry analysis, 1 × 10^6^ cells per well were seeded in a six‐well plate and treated with drugs as mentioned above. After 24 h, cells were washed with PBS, collected by centrifugation, stained with CRT primary antibody in the dark for 1 h and secondary antibody for 30 min, washed twice with PBS, and then analyzed for surface fluorescence signals by flow cytometry.

Additionally, CLSM was used to examine Hsp70 expression on the surface of 4T1 cells and the subcellular distribution of HMGB1. Similarly, 1 × 10^5^ cells per dish were seeded in a confocal dish and treated with drugs accordingly. After two washes with PBS, for Hsp70 detection, cells were blocked with goat serum for 1 h, then sequentially stained with Hsp70 primary antibody (ab181606, Abcam) for 1 h, secondary antibody (ab150077, Abcam) for 30 min, fixed, and stained with DAPI. For HMGB1 immunofluorescence staining, cells were fixed and permeabilized after drug incubation, blocked with goat serum for 1 h, and then incubated with Alexa Fluor 488‐conjugated anti‐HMGB1 antibody (ab195010, Abcam) overnight at 4°C. Cells were subsequently stained with DAPI and imaged by CLSM.

### 4.8 Detection of Oxygen Release by GB@vPCs (O_2_) in Cells

To investigate the effectiveness of GB@vPCs (O_2_) in releasing oxygen at the cellular level, we employed the [Ru(dpp)_3_]Cl_2_ fluorescent probe, which experiences fluorescence quenching upon encountering oxygen. Initially, 4T1 cells were seeded at a density of 1 × 10^5^ cells per well in a 12‐well plate. After cell adherence, they were cultivated under 37°C and 1% hypoxic conditions for 24 h to induce a hypoxic state. The culture medium was then replaced with fresh medium containing 10 µg mL^−1^ of [Ru(dpp)_3_]Cl_2_ fluorescent probe and cells were further incubated for 8 h. Thereafter, cells were allocated into different treatment groups, co‐cultured for 24 h with G@vPCs, GB@vPCs, G@vPCs (O_2_), GB@vPCs (O_2_), and ultrasound‐treated G@vPCs (O_2_) + US and GB@vPCs (O_2_) + US, respectively. All drug formulations contained GEM at a concentration of 0.04 µg mL^−1^. For ultrasound treatments, cells received ultrasound exposure after 8 h of incubation at settings of 1.0 MHz frequency, 1 W cm^−2^ power density, and 20% duty cycle for 5 min. Normoxic and hypoxic PBS groups served as controls. Following incubation, changes in intracellular oxygen levels were observed and evaluated via fluorescence microscopy; stronger red fluorescence correlated with higher degrees of hypoxia, conversely, reduced red fluorescence signified increased oxygen generation.

### 4.9 Analysis of HIF‐1α and P‐gp Protein Expression in 4T1 Cells Under Different Treatments

To explore variations in HIF‐1α and P‐gp protein levels in 4T1 cells under distinct conditions, Western Blotting was employed. The protocol entailed seeding 4T1 cells at a density of 1 × 10^6^ cells per well in a 6‐well plate. After attachment, cells were incubated under 37°C and 1% hypoxia for 24 h to induce hypoxia. Cells were then distributed into different groups for three replicates of identical treatments: co‐culture for 8 h with G@vPCs, GB@vPCs, GB@vPCs (O_2_), and ultrasound‐exposed GB@vPCs (O_2_) + US, all with GEM at 0.04 µg mL^−1^. For ultrasound treatments, cells were exposed after 4 h of incubation with parameters as before. Normoxic and hypoxic PBS groups were included as controls. Post‐treatments, cells were lysed in RIPA buffer with protease inhibitors on ice to extract total proteins, followed by protein quantification using a NanoDrop (Thermo Fisher Scientific). Protein samples were mixed with loading buffer containing β‐mercaptoethanol, heated at 95°C for 5 min, then centrifuged at 12 000 rpm for 2 min before loading. SDS‐PAGE was conducted on a 4%–20% gradient gel, and proteins were transferred to a 0.45 µm PVDF membrane at a constant current of 350 mA. After blocking with 5% skim milk, the membrane was incubated overnight at 4°C with primary antibodies against HIF‐1α (ab308433, Abcam), P‐gp (ab168337, Abcam), GAPDH and β‐actin. Following washing with PBST, the membrane was incubated with horseradish peroxidase‐conjugated secondary antibodies at room temperature for 1 h, and finally, the PVDF membrane was imaged after ECL development using a chemiluminescence detection system.

To assess the migratory and invasive capabilities of tumor cells, we employed Transwell assays. The preconditioning of cells followed a similar protocol to that used in the previous Western Blotting experiment. Initially, 4T1 cells were seeded at a density of 1 × 10^6^ cells per well in a 6‐well plate and incubated under hypoxic conditions for 24 h to induce a hypoxic state. Subsequently, cells were subjected to various drug treatments, including GB@vPCs, GB@vPCs (O_2_), and GB@vPCs (O_2_)+US, each for a duration of 8 h. For groups requiring ultrasound treatment, ultrasound intervention was administered as previously detailed after 4 h of incubation, followed by completion of the remaining 4 h incubation; the GEM concentration remained at 0.04 µg mL^−1^. Normoxic and hypoxic PBS groups served as controls. Following three rounds of identical treatments, 5 × 10^4^ cells from each treated 4T1 group were seeded onto the upper chambers of Transwells fitted with a polycarbonate membrane. For invasion assessment, the upper side of the polycarbonate membranes was coated with 50 µL of Matrigel (Corning, USA); no coating was applied for migration assessment. Serum‐free medium was added to the upper chamber, while the lower chamber received medium containing 10% FBS. After 24 h, cells on the upper surface of the membrane were gently scraped off, and cells that had migrated or invaded through the membrane were fixed with 4% paraformaldehyde. These cells were then stained with 0.1% crystal violet solution and visually inspected under an inverted light microscope.

### 4.10 Cell Cytotoxicity Assay

The cytotoxicity evaluation was carried out using the CCK‐8 assay. Initially, cells were seeded at a density of 8 × 10^3^ cells per well in a 96‐well plate and incubated overnight. The original medium was then replaced with fresh medium containing varying concentrations of vPCs, BMS1166, free GEM, G@vPCs, and GB@vPCs for 24 h, with GEM concentrations ranging from 0.0025 µg/ml to 1 µg mL^−1^, while other drug components matched the amounts present in GB@vPCs. Upon completion of incubation, 10% CCK‐8‐containing serum‐free medium was added to each well and further incubated at 37°C for another 1 h. Ultimately, absorbance values (OD) at a wavelength of 450 nm were measured for each well using a microplate reader.

To examine the impact of ultrasound treatment on cell safety, cells were seeded following the same protocol and divided into three groups: vPCs, vPCs+US, and vPCs (O_2_)+US, with vPCs concentrations ranging from 0.055 to 22 µg mL^−1^. At the 8‐hour mark, ultrasound treatment (using the same parameters as before) was applied to the designated groups, followed by the addition of CCK‐8 for cell viability assessment after 24 h as described earlier.

Moreover, cytotoxicity under hypoxic conditions was also investigated. Cells were seeded at a density of 5 × 10^3^ cells per well in a 96‐well plate and, after attachment, incubated under hypoxic conditions for 24 h to induce a hypoxic state. Cells were then treated with GB@vPCs, GB@vPCs (O_2_), or GB@vPCs (O_2_)+US, with GEM concentrations of 0.005, 0.01, 0.02, and 0.1 µg mL^−1^. Ultrasound treatment was administered at the 8‐hour point for the US groups, and 10% CCK‐8‐containing medium was introduced after 24 h of incubation. Following an additional 1 h of incubation at 37°C, the absorbance values at 450 nm were determined for all wells using a microplate reader.

To further evaluate the cytotoxicity of nanodroplets, a Calcein‐AM and PI double‐staining experiment was performed. Initially, cells were seeded at a density of 2 × 10^5^ cells per well in a 12‐well plate and incubated overnight. The original medium was then replaced with fresh medium containing various concentrations of vPCs, BMS1166, free GEM, G@vPCs, and GB@vPCs, with GEM at a concentration of 0.04 µg mL^−1^ and other drug components equivalent to those in GB@vPCs. After 24 h of drug‐cell incubation, cells were stained with Calcein‐AM and PI, washed with PBS, and visualized under an inverted fluorescence microscope where green indicated live cells and red represented dead cells.

For apoptosis assessment, an Annexin V‐FITC/PI double‐staining kit combined with flow cytometry was employed. Cells were seeded at a density of 2 × 10^5^ cells per well in a 12‐well plate and incubated overnight. The medium was exchanged with fresh medium containing vPCs, BMS1166, free GEM, G@vPCs, and GB@vPCs, with GEM at 0.04 µg mL^−1^ and other drugs in quantities matching GB@vPCs. After 24 h of incubation, cells were digested with trypsin without EDTA, collected by centrifugation, and resuspended in 500 µL buffer. Following Annexin V‐FITC and PI staining for 15 min, cells were analyzed by flow cytometry. Additionally, apoptosis induction under hypoxia was examined. Cells were seeded at 1.5 × 10^5^ cells per well, allowed to adhere, and then incubated under hypoxia for 24 h. Treatment with free GEM, G@vPCs, GB@vPCs, GB@vPCs (O_2_), and GB@vPCs (O_2_)+US (US treatment at 8 h as before) with GEM at 0.04 µg mL^−1^. Cells were collected, resuspended, stained, and analyzed similarly for apoptosis.

For exploring pyroptosis triggered by chemotherapy, cells were seeded and incubated as before with drugs. Pyroptosis was visually inspected under a fluorescence microscope, and supernatants were collected for lactate dehydrogenase (LDH) release measurement using an LDH test kit and interleukin‐1β (IL‐1β) secretion quantification by a mouse IL‐1β ELISA kit, both indicative of pyroptosis. Western blotting was further utilized to validate chemotherapy‐induced changes in pyroptosis‐related proteins. Cells were seeded at 1 × 10^6^ cells per well in a 6‐well plate and incubated overnight, then treated with free GEM at concentrations ranging from 0.02 to 0.10 µg mL^−1^ for 24 h. After treatment, cells were lysed, proteins extracted, and analyzed by Western blot following the previous protocol. For protein expression changes due to different drug treatments, a similar procedure was followed after incubating cells overnight, replacing media with drug‐containing media for 24 h, lysing cells, and conducting Western blot analysis accordingly.

### 4.11 Animal Experiments

Female BALB/c mice (6 weeks) were purchased from the Peking University Health Science Center Department of Laboratory Animal Science. All animal experiments were conducted in accordance with the ARRIVE guidelines. The study was approved by the Laboratory Animal Research Ethics Committee of Peking University Health Science Center and performed in compliance with all relevant ethical regulations (Approval No. A2022096).

### 4.12 In Vivo Biocompatibility Assessment

To investigate the in vivo biocompatibility of GB@vPCs(O_2_), we conducted evaluations through hemolysis tests and blood cell analysis. For Hemolysis Test Procedure: Blood was drawn from mice (1 mL) and centrifuged at 12 000 rpm for 5 min to isolate the red blood cell layer. These cells were then diluted in phosphate‐buffered saline (PBS) to a 4% concentration for later use. Sequentially, GB@vPCs(O_2_) at varying lipid concentrations (31.3, 62.5, 125, 250, and 500 µg mL^−1^) were each mixed with the 4% red blood cell PBS suspension, with both the nanoparticle‐containing PBS and the 4% red cell PBS solution measuring 500 µL per group. Control groups were also established, including a negative control (4% red blood cell PBS mixed with an equal volume of PBS) and a positive control (4% red blood cell PBS mixed with an equal volume of deionized water). After incubating these mixtures at 37°C for 4 h, they were centrifuged again at 12 000 rpm for 5 min. The tubes were photographed afterward, and the supernatant was collected to measure its absorbance at 570 nm using a microplate reader.

For Blood Cell Analysis Procedure: Mice were administered GB@vPCs(O_2_) nanodroplets at a dose of 2.5 mg kg^−1^ GEM via tail vein injection. Blood routine tests were conducted multiple times, including pre‐injection and on days 1, 7, 14, and 28 post‐injection. Key parameters in the blood count analysis comprised Red Blood Cell Count (RBC), White Blood Cell Count (WBC), Platelet Count (PLT), Mean Corpuscular Volume (MCV), Hemoglobin Content (HGB), Mean Corpuscular Hemoglobin (MCH), Mean Corpuscular Hemoglobin Concentration (MCHC), and Hematocrit (HCT).

### 4.13 Plasma Pharmacokinetics Measurement

To examine the plasma clearance rate of nanodroplets in mice, DSPE‐Cy5.5 was first used to fluorescently label GB@vPCs and GB@vPLs. Following this, labeled nanodroplets were intravenously injected into healthy female BALB/c mice at a gemcitabine (GEM) dose of 2.5 mg kg^−1^ via the tail vein. At several predetermined time points, blood samples were collected from the retro‐orbital plexus of the mice using heparin‐coated capillaries, and promptly placed on ice to maintain stability. High‐speed centrifugation (12 000 rpm for 5 min) was then employed to separate the plasma fraction. Thereafter, the plasma samples obtained at each time point were subjected to Cy5.5 fluorescence detection on a microplate reader, with excitation set at 660 nm and emission at 710 nm. The recorded data were utilized to plot the curve representing the change in relative Cy5.5 concentration over time. For pharmacokinetic analysis, GraphPad software was employed, fitting the curve to a dual exponential decay model (ExpDec2) to ascertain the plasma elimination half‐life (t_1/2_).

### 4.14 In Vivo Near‐Infrared Fluorescence Imaging

Employing the IVIS Spectrum small animal in vivo optical imaging system, we evaluated the in vivo fluorescence distribution of nanodroplets labeled with DSPE‐Cy5.5, including GB@vPCs, GB@vPLs, and GB@PCs. Using a 4T1 tumor‐bearing mouse model, 200 µL of the nanodroplet suspensions (containing a gemcitabine (GEM) dose of 2.5 mg kg^−1^) were administered via intravenous injection. Following injection, mice were subjected to in vivo fluorescence imaging at multiple time points including 1, 4, 8, 12, 24, 36, 48, 72, 96, and 120 h to capture Cy5.5 fluorescence signals, thereby tracing the biodistribution of the nano‐drugs within the body. When reaching 120 h, mice were euthanized, and vital organs including the heart, liver, spleen, lungs, kidneys, and tumors were harvested. These organs were then laid flat on black paper and imaged for their fluorescence to observe the accumulation and distribution of nanodroplets in key organs and tumor sites.

### 4.15 Ultrasound Imaging

To evaluate the contrast effect of nanodroplets in ultrasonography, studies were conducted both in vitro and in vivo. Initially, an in vitro imaging experiment was performed where 1 mL of GB@vPCs solutions at various concentrations (0.2, 0.5, 1, and 2 mg mL^−1^) were infused into latex tubes and placed in a petri dish filled with degassed deionized water. The VINNO70 ultrasound system was then used to image the latex tubes (MI 0.09/ TIS 0.1, AP 50%, 3 MHz). To better understand the influence of ultrasound irradiation (1 MHz, 1 W cm^−2^, 20‐50% duty cycle) and oxygenation on imaging outcomes, GB@vPCs were imaged under both oxygenated and non‐oxygenated states. Baseline ultrasound images were captured, after which GB@vPCs and oxygenated GB@vPCs(O_2_) were removed from the latex tubes and placed in a syringe for ultrasound exposure. The ultrasound parameters were set to a frequency of 1.0 MHz, power density of 1 W cm^−2^, and duty cycle of 20%, for 5 min. Ultrasound imaging was repeated post‐exposure to compare the contrast effects before and after ultrasound irradiation. For in vivo contrast effect assessment, oxygenated GB@vPCs (O_2_) were first injected directly into the tumors of mice via intratumoral injection, and the contrast enhancement was observed and recorded using the VINNO70 ultrasound system. Further investigation into the contrast performance via intravenous administration involved injecting 200 µL of GB@vPCs (O_2_) into 4T1 tumor‐bearing mice. Before injection and at 6, 12, 24, and 48 h post‐injection, the tumors in the mice were imaged using the VINNO70 ultrasound system. At the 48‐hour mark, the tumors were subjected to ultrasound irradiation with settings adjusted to a frequency of 1.0 MHz, power density of 1 W cm^−2^, duty cycle of 50%, for 5 min. Subsequent ultrasound imaging was performed to clarify the impact of ultrasound irradiation on the in vivo contrast effect of nanodroplets.

### 4.16 Photoacoustic Imaging

To investigate the efficacy of oxygenated nanodroplets, GB@vPCs (O_2_), in relieving tumor hypoxia in mice, in vivo three‐dimensional photoacoustic imaging of tumor blood oxygen saturation was performed. Initially, oxygenated GB@vPCs (O_2_) were intravenously injected into the mice. Before injection and at 24 and 48 h post‐injection, three‐dimensional multispectral photoacoustic imaging was carried out, capturing tumor images at 20‐nm wavelength intervals across the range of 700 to 990 nm using a photoacoustic imaging system. Leveraging the standard optical absorption spectra of oxyhemoglobin and deoxyhemoglobin, tumor blood oxygen saturation (SO_2_) was calculated using a standard linear unmixing method. Following the 48‐hour imaging session, the tumors in the mice underwent ultrasound treatment with parameters set at a frequency of 1.0 MHz, power density of 1 W cm^−2^, and duty cycle of 50%, for a duration of 5 min. Photoacoustic imaging was repeated post‐ultrasound exposure. To verify whether ultrasound exposure itself directly affected tumor blood oxygen saturation, a control experiment was conducted on mice without drug injection, undergoing photoacoustic imaging before and after ultrasound exposure, comparing the changes in blood oxygenation under ultrasound intervention.

### 4.17 Immunotherapy Timing Optimization

To study the in vivo effect of chemotherapy drugs on PD‐L1 expression on the surface of tumor cells, a 4T1 breast cancer mouse model was established. Specifically, log‐phase 4T1 cells were harvested, and resuspended in PBS, and a 100 µL suspension containing 2 × 10^6^ cells was subcutaneously injected into the right flank of 6‐to‐8‐week‐old female BALB/c mice to promote tumor growth. When tumors reached approximately 100 mm^3^ in size, G@vPCs were injected intravenously. Tumor tissues were collected at 12, 24, 48, and 72 h post‐injection, fixed in 10% formalin, embedded in paraffin, and PD‐L1 expression was assessed by immunofluorescence staining.

To investigate the effect of timing of ultrasound‐triggered immune therapy drug release on systemic immune responses, tumors were sonicated at the same time points (12, 24, 48, and 72 h post‐injection) with ultrasound parameters set at 1.0 MHz frequency, 1 W cm^−2^ power density, 50% duty cycle for 5 min. Two days after sonication, draining lymph nodes were collected, homogenized, and dendritic cells (DCs) were isolated. T cells were isolated from tumor single‐cell suspensions. The isolated DCs and T cells were incubated with purified anti‐mouse CD16/CD32 antibody for 10–20 min prior to staining. DCs were stained with anti‐CD11c‐PE, anti‐CD86‐PE‐Cy7, and anti‐CD80‐APC antibodies and analyzed by flow cytometry for maturation status. T lymphocyte subsets within tumors were stained with anti‐CD3‐APC, anti‐CD4‐PE, and anti‐CD8a‐FITC antibodies, and analyzed by flow cytometry.

Detailed gating strategies for all flow cytometry experiments are provided in the Supporting Information (Figures S18–S20). Sera were also collected and levels of TNF‐α, IFN‐γ, and IL‐1β in mice sera were detected using ELISA kits to evaluate systemic inflammatory response.

### 4.18 In Vivo Primary Tumor Treatment Evaluation

To systematically assess the in vivo anti‐tumor therapeutic effect of nanodroplets, a 4T1 breast cancer model was established in mice. When tumors reached about 100 mm^3^, mice were randomly divided into treatment groups (5 mice/group): 1) PBS, 2) vPCs(O_2_) + US, 3) B@vPCs + US, 4) G@vPCs, 5) GB@vPCs, 6) GB@vPLs, 7) GB@vPCs(O_2_), 8) GB@vPCs(O_2_) + US. Mice were treated on days 0, 5, and 9. For ultrasound groups, tumors were sonicated 48 h after injection (day 2, 7, 11) under the same ultrasound parameters. Tumor volumes and body weights were recorded every two days. In the end, mice were euthanized, tumors were photographed and weighed, and vital organs were collected for histopathological examination. Sera were tested for liver and kidney function. On day 13, tumors were analyzed pathologically, including H&E staining, Ki‐67 for proliferation, TUNEL for apoptosis, and immunofluorescence for PD‐L1, HIF‐1α, IL‐1β, CD4, CD8, etc.

Draining lymph nodes were also collected for immune response analysis. DCs were isolated, stained with antibodies, and analyzed by flow cytometry. Tumor single‐cell suspensions were stained for T lymphocyte subset analysis. Sera were assayed for TNF‐α, IFN‐γ, and IL‐1β.

### 4.19 In Vivo Distant Tumor Treatment Assessment

To evaluate distant tumor suppression, a primary 4T1 tumor was first established on the right flank of mice, with a distant tumor created on the left flank on day 5. The primary tumor was treated as previously described. Mice were euthanized, distant tumors photographed, and volumes measured every two days. On day 3 after the third treatment, distant tumors were processed for H&E, Ki‐67, TUNEL, CD4, and CD8 staining. Lymphocyte infiltration was assessed similarly.

### 4.20 Lung Metastasis and Tumor Recurrence Study

For lung metastasis, a primary 4T1 tumor model was first established, followed by intravenous injection of 4T1 cells to simulate pulmonary metastasis. Mice were treated with PBS, GB@vPCs(O_2_), or GB@vPCs(O_2_) + US following a procedure similar to that described above and were euthanized on day 3 after the final treatment. Lungs were collected for gross observation, H&E staining, and immunofluorescence analysis.

For the recurrence model, primary tumors were treated with GB@vPCs(O_2_) + US. A secondary tumor was then inoculated on the contralateral side on day 3 after the final treatment, and mouse spleens were analyzed for immune memory after tumor recurrence. These experiments collectively evaluated the nanodroplet therapy against primary, distant, metastatic, and recurrent tumors, incorporating immunological and pathological assessments.

### 4.21 Statistical Analysis

Experimental results are presented as mean values plus or minus the standard deviation. After statistical analysis using GraphPad Prism version 8.0.2 software, the data are plotted into statistical graphs. For comparisons among multiple groups, one‐way or two‐way ANOVA followed by Bonferroni's or Tukey's multiple comparisons tests were applied. Differences are considered statistically significant when *p < 0.05 and highly significant when **p < 0.01.

## Author Contributions

Suhui Sun and Ruiqi Wu served as co–first authors and contributed equally to this work. Suhui Sun performed the experiments, analyzed the data, and drafted the manuscript. Ruiqi Wu performed the experiments and analyzed the data. Qingshuang Tang, Yunli Xu, Wanrui Shi, Mengxin Wang, and Haonan Wang analyzed the data. Yan Luo and Cheng Ma performed the photoacoustic imaging. Xiaolong Liang conceived the idea, supervised the study, interpreted the results, and revised the manuscript. All authors contributed to the critical reading of and commented on the manuscript, helped to interpret the data, and approved the final manuscript.

## Conflicts of Interest

The authors declare no conflicts of interest.

## Supporting information




**Supporting File**: advs77722‐sup‐0001‐SuppMat.docx

## Data Availability

The data that support the findings of this study are available from the corresponding author upon reasonable request.

## References

[1] R. L. Siegel , K. D. Miller , and A. Jemal , “Cancer Statistics, 2020,” CA: A Cancer Journal for Clinicians 70, no. 1 (2020): 7–30, 10.3322/caac.21590. 31912902

[2] W. H. Fridman , F. Pages , C. Sautes‐Fridman , and J. Galon , “The Immune Contexture In Human Tumours: Impact on Clinical Outcome,” Nature Reviews Cancer 12, no. 4 (2012): 298–306, 10.1038/nrc3245. 22419253

[3] P. Sharma and J. P. Allison , “The Future of Immune Checkpoint Therapy,” Science 348, no. 6230 (2015): 56–61, 10.1126/science.aaa8172. 25838373

[4] C. Robert , J. Schachter , G. V. Long , et al., “Pembrolizumab versus Ipilimumab in Advanced Melanoma,” New England Journal of Medicine 372, no. 26 (2015): 2521–2532, 10.1056/NEJMoa1503093. 25891173

[5] P. C. Tumeh , C. L. Harview , J. H. Yearley , et al., “PD‐1 Blockade Induces Responses by Inhibiting Adaptive Immune Resistance,” Nature 515, no. 7528 (2014): 568–571, 10.1038/nature13954. 25428505 PMC4246418

[6] R. S. Herbst , J. C. Soria , M. Kowanetz , et al., “Predictive Correlates Of Response to the Anti‐PD‐L1 Antibody MPDL3280A in Cancer Patients,” Nature 515, no. 7528 (2014): 563–567, 10.1038/nature14011. 25428504 PMC4836193

[7] J. D. Wolchok , V. Chiarion‐Sileni , R. Gonzalez , et al., “Overall Survival With Combined Nivolumab and Ipilimumab in Advanced Melanoma,” New England Journal of Medicine 377, no. 14 (2017): 1345–1356, 10.1056/NEJMoa1709684. 28889792 PMC5706778

[8] A. H. Sharpe and K. E. Pauken , “The Diverse Functions of the PD1 Inhibitory Pathway,” Nature Reviews Immunology 18, no. 3 (2018): 153–167, 10.1038/nri.2017.108. 28990585

[9] E. B. Garon , N. A. Rizvi , R. Hui , et al., “Pembrolizumab for the Treatment of Non–Small‐Cell Lung Cancer,” New England Journal of Medicine 372, no. 21 (2015): 2018–2028, 10.1056/NEJMoa1501824. 25891174

[10] R. Zappasodi , T. Merghoub , and J. D. Wolchok , “Emerging Concepts for Immune Checkpoint Blockade‐Based Combination Therapies,” Cancer Cell 34, no. 4 (2018): 690, 10.1016/j.ccell.2018.09.008. 30300584 PMC6222254

[11] J. E. Rosenberg , J. Hoffman‐Censits , T. Powles , et al., “Atezolizumab in Patients With Locally Advanced And Metastatic Urothelial Carcinoma Who Have Progressed Following Treatment With Platinum‐Based Chemotherapy: A Single‐Arm, Multicentre, Phase 2 Trial,” The Lancet 387, no. 10031 (2016): 1909–1920, 10.1016/S0140-6736(16)00561-4. PMC5480242 26952546

[12] P. S. Hegde , V. Karanikas , and S. Evers , “The Where, the When, and the How of Immune Monitoring for Cancer Immunotherapies in the Era of Checkpoint Inhibition,” Clinical Cancer Research 22, no. 8 (2016): 1865–1874, 10.1158/1078-0432.Ccr-15-1507. 27084740

[13] T. F. Gajewski , H. Schreiber , and Y. X. Fu , “Innate and Adaptive Immune Cells in the Tumor Microenvironment,” Nature Immunology 14, no. 10 (2013): 1014–1022, 10.1038/ni.2703. 24048123 PMC4118725

[14] C. Wang , J. Wang , X. Zhang , et al., “In Situ Formed Reactive Oxygen Species–Responsive Scaffold With Gemcitabine and Checkpoint Inhibitor for Combination Therapy,” Science Translational Medicine 10, no. 429 (2018): eaan3682, 10.1126/scitranslmed.aan3682. 29467299

[15] D. Fukumura , J. Kloepper , Z. Amoozgar , D. G. Duda , and R. K. Jain , “Enhancing Cancer Immunotherapy Using Antiangiogenics: Opportunities and Challenges,” Nature Reviews Clinical Oncology 15, no. 5 (2018): 325–340, 10.1038/nrclinonc.2018.29. PMC5921900 29508855

[16] A. Johansson‐Percival , B. He , Z. J. Li , et al., “De Novo Induction Of Intratumoral Lymphoid Structures And Vessel Normalization Enhances Immunotherapy In Resistant Tumors,” Nature Immunology 18, no. 11 (2017): 1207–1217, 10.1038/ni.3836. 28892469

[17] H. Yuan , W. Jiang , C. A. von Roemeling , et al., “Multivalent Bi‐Specific Nanobioconjugate Engager For Targeted Cancer Immunotherapy,” Nature Nanotechnology 12, no. 8 (2017): 763–769, 10.1038/nnano.2017.69. 28459470

[18] T. T. Smith , S. B. Stephan , H. F. Moffett , et al., “In Situ Programming Of Leukaemia‐Specific T Cells Using Synthetic DNA Nanocarriers,” Nature Nanotechnology 12, no. 8 (2017): 813–820, 10.1038/nnano.2017.57. PMC5646367 28416815

[19] S. G. Awuah , Y. R. Zheng , P. M. Bruno , M. T. Hemann , and S. J. Lippard , “A Pt(IV) Pro‐drug Preferentially Targets Indoleamine‐2,3‐dioxygenase, Providing Enhanced Ovarian Cancer Immuno‐Chemotherapy,” Journal of the American Chemical Society 137, no. 47 (2015): 14854–14857, 10.1021/jacs.5b10182. 26561720 PMC4772771

[20] C. He , X. Duan , N. Guo , et al., “Core‐Shell Nanoscale Coordination Polymers Combine Chemotherapy And Photodynamic Therapy To Potentiate Checkpoint Blockade Cancer Immunotherapy,” Nature Communications 7, no. 1 (2016): 12499, 10.1038/ncomms12499. PMC4992065 27530650

[21] M. Roselli , V. Cereda , M. G. di Bari , et al., “Effects of Conventional Therapeutic Interventions on the Number and Function Of Regulatory T Cells,” Oncoimmunology 2 (2013): 27025, 10.4161/onci.27025. PMC3862634 24353914

[22] D. S. Ettinger , D. E. Wood , D. L. Aisner , et al., “Non–Small Cell Lung Cancer, Version 3.2022, NCCN Clinical Practice Guidelines In Oncology,” Journal of the National Comprehensive Cancer Network 20, no. 5 (2022): 497, 10.6004/jnccn.2022.0025. 35545176

[23] G. Kroemer , L. Galluzzi , O. Kepp , and L. Zitvogel , “Immunogenic Cell Death in Cancer Therapy,” Annual Review of Immunology 31, no. 1 (2013): 51–72, 10.1146/annurev-immunol-032712-100008. 23157435

[24] P. Kadiyala , D. Li , F. M. Nunez , et al., “High Density Lipoprotein‐Mimicking Nanodiscs For Chemo‐Immunotherapy Against Glioblastoma Multiforme,” ACS Nano 13, no. 2 (2019): 1365, 10.1021/acsnano.8b06842. 30721028 PMC6484828

[25] S. Sun , Q. Tang , Y. Wang , et al., “In Situ Micro–Nano Conversion Augmented Tumor‐Localized Immunochemotherapy,” ACS AppliedMaterials & Interfaces 14, no. 23 (2022): 27013, 10.1021/acsami.2c02490. 35657950

[26] S. H. Sun , R. Q. Wu , S. S. Ma , et al., “Stable Liposomal Cerasome For Ultrasound/H_2_O_2_‐Activated CO Release To Augment Tumor Chemotherapy And Immunotherapy,” Chemical Engineering Journal 510 (2025): 161398, 10.1016/j.cej.2025.161398.

[27] L. Bezu , A. Sauvat , J. Humeau , et al., “eIF2α Phosphorylation is Pathognomonic for Immunogenic Cell Death,” Cell Death & Differentiation 25, no. 8 (2018): 1375–1393, 10.1038/s41418-017-0044-9. 29358668 PMC6113215

[28] E. B. Nejad , T. C. van der Sluis , S. van Duikeren , et al., “Tumor Eradication by Cisplatin Is Sustained by CD80/86‐Mediated Costimulation of CD8+ T Cells,” Cancer Research 76, no. 20 (2016): 6017–6029, 10.1158/0008-5472.Can-16-0881. 27569212

[29] Q. Gao , S. Wang , F. Li , et al., “High Mobility Group Protein B1 Decreases Surface Localization of PD‐1 to Augment T‐cell Activation,” Cancer Immunology Research 10, no. 7 (2022): 844, 10.1158/2326-6066.CIR-21-0652. 35580259

[30] Y. Barenholz , “Doxil® — The first FDA‐approved nano‐drug: Lessons learned,” Journal of Controlled Release 160, no. 2 (2012): 117–134, 10.1016/j.jconrel.2012.03.020. 22484195

[31] G. Pillai and M. L. Ceballos‐Coronel , “Science and Technology Of The Emerging Nanomedicines In Cancer Therapy: A Primer For Physicians And Pharmacists,” SAGE Open Medicine 1 (2013): 2050312113513759, 10.1177/2050312113513759. 26770691 PMC4687778

[32] F. Man , T. Lammers , and R. T M de Rosales , “Imaging Nanomedicine‐Based Drug Delivery: A Review of Clinical Studies,” Molecular Imaging and Biology 20, no. 5 (2018): 683–695, 10.1007/s11307-018-1255-2. 30084044 PMC6139024

[33] V. P. Torchilin , “Recent Advances With Liposomes As Pharmaceutical Carriers,” Nature Reviews Drug Discovery 4, no. 2 (2005): 145–160, 10.1038/nrd1632. 15688077

[34] T. O. B. Olusanya , R. R. Haj Ahmad , D. M. Ibegbu , J. R. Smith , and A. A. Elkordy , “Liposomal Drug Delivery Systems And Anticancer Drugs,” Molecules 23, no. 4 (2018): 907, 10.3390/molecules23040907. 29662019 PMC6017847

[35] Y. Li , R. Liu , J. Yang , et al., “Enhanced Retention And Anti‐Tumor Efficacy Of Liposomes By Changing Their Cellular Uptake And Pharmacokinetics Behavior,” Biomaterials 41 (2015): 1–4, 10.1016/j.biomaterials.2014.11.010. 25522960

[36] P. Yingchoncharoen , D. S. Kalinowski , and D. R. Richardson , “Lipid‐Based Drug Delivery Systems in Cancer Therapy: What Is Available and What Is Yet to Come,” Pharmacological Reviews 68, no. 3 (2016): 701–787, 10.1124/pr.115.012070. 27363439 PMC4931871

[37] J. J. Mittag , B. Kneidl , T. Preiβ , et al., “Impact of Plasma Protein Binding On Cargo Release by Thermosensitive Liposomes Probed By Fluorescence Correlation Spectroscopy,” European Journal of Pharmaceutics and Biopharmaceutics 119 (2017): 215–223, 10.1016/j.ejpb.2017.06.022. 28648864

[38] M. Kogan , B. Feng , B. Nordén , S. Rocha , and T. Beke‐Somfai , “Shear‐Induced Membrane Fusion in Viscous Solutions,” Langmuir 30, no. 17 (2014): 4875–4878, 10.1021/la404857r. 24758573

[39] A. L. Bernard , M. A. Guedeau‐Boudeville , V. Marchi‐Artzner , T. Gulik‐Krzywicki , J. M. di Meglio , and L. Jullien , “Shear‐Induced Permeation And Fusion Of Lipid Vesicles,” Journal of Colloid and Interface Science 287, no. 1 (2005): 298–306, 10.1016/j.jcis.2004.12.019. 15914177

[40] A. H. Sharpe , E. J. Wherry , R. Ahmed , and G. J. Freeman , “The Function Of Programmed Cell Death 1 And Its Ligands In Regulating Autoimmunity And Infection,” Nature Immunology 8, no. 3 (2007): 239–245, 10.1038/ni1443. 17304234

[41] L. Chen and X. Han , “Anti–PD‐1/PD‐L1 Therapy of Human Cancer: Past, Present, and Future,” Journal of Clinical Investigation 125, no. 9 (2015): 3384–3391, 10.1172/jci80011. 26325035 PMC4588282

[42] J. S. Weber , K. C. Kähler , and A. Hauschild , “Management of Immune‐Related Adverse Events and Kinetics of Response With Ipilimumab,” Journal of Clinical Oncology 30, no. 21 (2012): 2691–2697, 10.1200/JCO.2012.41.6750. 22614989

[43] J. Naidoo , D. B. Page , B. T. Li , et al., “Toxicities of the anti‐PD‐1 and anti‐PD‐L1 Immune Checkpoint Antibodies,” Annals of Oncology 26, no. 12 (2015): 2375–2391, 10.1093/annonc/mdv383. 26371282 PMC6267867

[44] R. S. Riley , C. H. June , R. Langer , and M. J. Mitchell , “Delivery Technologies For Cancer Immunotherapy,” Nature Reviews Drug Discovery 18, no. 3 (2019): 175–196, 10.1038/s41573-018-0006-z. 30622344 PMC6410566

[45] J. Peng , Y. Xiao , W. Li , et al., “Photosensitizer Micelles Together With IDO Inhibitor Enhance Cancer Photothermal Therapy And Immunotherapy,” Advanced Science (Weinh) 5, no. 5 (2018): 1700891, 10.1002/advs.201700891. PMC5979747 29876215

[46] J. L. Adams , J. Smothers , R. Srinivasan , and A. Hoos , “Big Opportunities for Small Molecules In Immuno‐Oncology,” Nature Reviews Drug Discovery 14, no. 9 (2015): 603–622, 10.1038/nrd4596. 26228631

[47] W. Yao , X. Zhao , Y. Gong , et al., “Impact of the Combined Timing of PD‐1/PD‐L1 Inhibitors And Chemotherapy on the Outcomes in Patients With Refractory Lung Cancer,” ESMO Open 6, no. 2 (2021): 100094, 10.1016/j.esmoop.2021.100094. 33780892 PMC8041717

[48] W. Xing , L. Zhao , Y. Zheng , et al., “The Sequence Of Chemotherapy And Toripalimab Might Influence The Efficacy Of Neoadjuvant Chemoimmunotherapy In Locally Advanced Esophageal Squamous Cell Cancer—A Phase II Study,” Frontiers in Immunology 12 (2021): 772450, 10.3389/fimmu.2021.772450. 34938292 PMC8685246

[49] C. Zhu , Y. Shi , Q. Li , et al., “Rational Administration Sequencing Of Immunochemotherapy Elicits Powerful Anti‐Tumor Effect,” Journal of Controlled Release 341 (2022): 769–781, 10.1016/j.jconrel.2021.12.022. 34952044

[50] M. Wu , Q. Huang , Y. Xie , et al., “Improvement of the Anticancer Efficacy of PD‐1/PD‐L1 Blockade via Combination Therapy and PD‐L1 Regulation,” Journal of hematology & oncology 15, no. 1 (2022): 24, 10.1186/s13045-022-01242-2. 35279217 PMC8917703

[51] S. H. Sun , S. J. Sun , Y. Sun , et al., “Bubble‐Manipulated Local Drug Release From A Smart Thermosensitive Cerasome For Dual‐Mode Imaging Guided Tumor Chemo‐Photothermal Therapy,” Theranostics 9, no. 26 (2019): 8138, 10.7150/thno.36762. 31754386 PMC6857040

[52] X. Ma , M. Yao , J. Shi , et al., “High Intensity Focused Ultrasound‐Responsive and Ultrastable Cerasomal Perfluorocarbon Nanodroplets for Alleviating Tumor Multidrug Resistance and Epithelial–Mesenchymal Transition,” ACS Nano 14, no. 11 (2020): 15904–15918, 10.1021/acsnano.0c07287. 33175487

[53] X. L. Liang , J. Gao , L. D. Jiang , et al., “Nanohybrid Liposomal Cerasomes With Good Physiological Stability And Rapid Temperature Responsiveness For High Intensity Focused Ultrasound Triggered Local Chemotherapy Of Cancer,” ACS Nano 9, no. 2 (2015): 1280, 10.1021/nn507482w. 25599568

[54] R. Wu , Y. Wang , S. Sun , et al., “Ultrasound Triggered Local Sequential Reactive Oxygen Species And Nitric Oxide Release For Enhanced Cerasomal Drug Delivery,” Chemical Engineering Journal 486 (2024): 150134, 10.1016/j.cej.2024.150134.

[55] J. Zhang , L. Sun , L. Jiang , et al., “Regulation of CTLs/tregs via Highly Stable And Ultrasound‐Responsive Cerasomal Nano‐Modulators For Enhanced Colorectal Cancer Immunotherapy,” Advanced Science (Weinh) 11, no. 22 (2024): 2400485, 10.1002/advs.202400485. PMC11165532 38552151

[56] Q. Ma , S. Wu , L. Yang , et al., “Hyaluronic Acid‐Guided Cerasome Nano‐Agents For Simultaneous Imaging And Treatment Of Advanced Atherosclerosis,” Advanced Science (Weinh) 10, no. 5 (2023): 2202416, 10.1002/advs.202202416. PMC9929131 36529695

[57] C. Du , R. Wu , W. Yan , et al., “Ultrasound‐Controlled Delivery Of Growth Factor‐Loaded Cerasomes Combined With Polycaprolactone Scaffolds Seeded With Bone Marrow Mesenchymal Stem Cells For Biomimetic Tendon‐To‐Bone Interface Engineering,” ACS Applied Materials &Interfaces 16, no. 1 (2024): 292–304, 10.1021/acsami.3c14959. 38133932 PMC10789257

[58] J. Yu , S. He , C. Zhang , et al., “Polymeric STING Pro‐agonists for Tumor‐Specific Sonodynamic Immunotherapy,” Angewandte Chemie 62, no. 32 (2023): 202307272, 10.1002/anie.202307272. 37312610

[59] L. Yin , F. Yang , W. Wang , et al., “PSMA‐Targeted Nanoparticles With PI3K/mTOR Dual Inhibitor Downregulate P‐glycoprotein and Inactivate Myeloid‐Derived Suppressor Cells For Enhanced Chemotherapy And Immunotherapy In Prostate Cancer,” Advanced Materials 37, no. 26 (2025): 2415322, 10.1002/adma.202415322. 40255060

[60] T. G. Nguyen Cao , Q. T. Hoang , J. H. Kang , et al., “Bioreducible Exosomes Encapsulating Glycolysis Inhibitors Potentiate Mitochondria‐Targeted Sonodynamic Cancer Therapy Via Cancer‐Targeted Drug Release And Cellular Energy Depletion,” Biomaterials 301 (2023): 122242, 10.1016/j.biomaterials.2023.122242. 37473534

[61] H. Liao , Y. Xiong , J. Li , et al., “GSH‐Triggered Nitric Oxide‐Releasing Polycarbonate Nanoplatform for Synergistic Gas‐Sonodynamic Antitumor Therapy,” Biomacromolecules 26, no. 11 (2025): 7632, 10.1021/acs.biomac.5c01149. 41051211

[62] X. Liang , X. Li , L. Jing , et al., “Design and Synthesis of Lipidic Organoalkoxysilanes for the Self‐Assembly of Liposomal Nanohybrid Cerasomes With Controlled Drug Release Properties,” Chemistry—A European Journal 19, no. 47 (2013): 16113–16121, 10.1002/chem.201302518. 24123292

[63] Y. Song , S. Xu , J. Zhang , et al., “Ultrasound‐Mediated Piezocatalysis Triggers NO Release to Augment Targeted Immunotherapy of Pancreatic Cancer,” ACS Nano 19, no. 36 (2025): 32654–32673, 10.1021/acsnano.5c10301. 40905947

[64] Q. Zhang , Q. Liu , J. Liu , et al., “Integrating PD‐L1‐Targeted Radioligand With Protein Degradation For Precision Tumor Theranostics,” Journal of Controlled Release 397 (2026): 115145, 10.1016/j.jconrel.2026.115145. 42372826

[65] E. Teixidor‐Vilà , J. A. Encinar , B. Martin‐Castillo , et al., “Silibinin is a Natural Molecular Glue that Inhibits PD‐L1 Glycomaturation,” Phytomedicine 159 (2026): 158481, 10.1016/j.phymed.2026.158481. 42365692

