# Supplementary material for: Spatiotemporally Controlled Sequential Chemo‐Immunotherapy Activates Pyroptosis for Robust Anti‐Tumor Immunotherapy
Source: Adv Sci (Weinh). 2026 Sep 16:e77722. Online ahead of print. doi: 10.1002/advs.77722 (PMC13583098; doi:10.1002/advs.77722)
Supplement: Supplementary file 1 [file ADVS-9999-e77722-s001.docx]

**Supporting Information**

**Spatiotemporally Controlled Sequential Chemo-Immunotherapy Activates Pyroptosis for Robust Anti-Tumor Immunotherapy**

*Suhui Sun^1,2,3^ Ruiqi Wu^1,2,4^, Qingshuang Tang^1,2^,* *Yunli Xu^1,2^,* *Wanrui Shi^1,2^, Mengxin Wang^1,2^, Haonan Wang^1,2^, Yan Luo^5^, Cheng Ma^5^*, *Xiaolong Liang*^1,2^*

^1^ State Key Laboratory of Vascular Homeostasis and Remodeling, Department of Ultrasound, Peking University Third Hospital, Beijing 100191, China.

^2^ Beijing Key Laboratory of Interdisciplinary Research in Gastrointestinal Oncology (BLGO), Peking University Third Hospital, Beijing 100191, China.

^3^ General Ultrasound Department, Beijing Anzhen Hospital, Capital Medical University, Beijing 100029, China.

^4^ Department of Ultrasound, The First Affiliated Hospital of Nanchang University, Nanchang, 330006, China.

^5^ Department of Electronic Engineering, Tsinghua University, Beijing 100084, P. R. China.

*To whom correspondence should be addressed

E-mail: Xiaolong Liang: [xiaolong_liang@bjmu.edu.cn](mailto:xiaolong_liang@bjmu.edu.cn)

**Table S1.** Molar ratios of components in different formulations of GB@vPCs and GB@vPLs

| **Formulations** | **GEM-Lip** | **CFL/DSPC** | **DSPE-PEG-Var7** | **DSPE-PEG2000** |
| --- | --- | --- | --- | --- |
| **GB@vPCs** | **20** | **65** | **5** | **10** |
| **GB@vPCs** | **30** | **55** | **5** | **10** |
| **GB@vPCs** | **40** | **45** | **5** | **10** |
| **GB@vPCs** | **50** | **35** | **5** | **10** |
| **GB@vPLs** | **30** | **55** | **5** | **10** |

**Figure S1.** The synthetic route of GEM-Lip, along with the mechanism for the release of gemcitabine.

**Figure S2.** Mass Spectra of Compound **2**.

**Figure S3.** Mass Spectra of GEM-Lip.

**Figure S4.** Mass Spectrometry Analysis of DSPE-PEG-Var7.

**Figure S5.** Variation in encapsulation efficiency and drug loading content of GB@vPCs under different mass feeding ratios of BMS1166.

**Figure S6.** Stability of GB@vPCs at 37 °C over 7 days. (a) Particle size distribution, (b) average diameter, (c) zeta potential, and (d) summary of particle size, zeta potential, and PDI on days 1, 3, and 7.

**Figure S7.** DLS size distribution of GB@vPCs after ultrasound treatment.

**Figure S8.** Fourier-transform infrared spectra of Cerasome and GB@vPCs.

**Figure S9.** Quantification of flow cytometry from Figure 3b, presented as mean ± SD (n = 3).

**Figure S10.** Flow cytometry of CRT exposure on the surface of 4T1 cells following various treatments.

**Figure S11.** Quantification of invaded cells from Figure 4e. Data are presented as mean ± SD (n = 3)

**Figure S12.** Viability of 4T1 cells after treatment with vPCs, vPCs+US, and vPCs (O_2_) +US, assessed by the CCK-8 assay.

**Figure S13.** Quantitative flow cytometry analysis of apoptosis in 4T1 cells.

**Figure S14.** a) Hemolysis rate and corresponding images of the hemolytic assay after incubation of RBCs with GB@vPCs(O_2_) at varying concentrations. b) Hematology analyses of mice before injection and on days 1, 7, 14, and 28 following intravenous administration of GB@vPCs(O_2_).

**Figure S15**. a) Ultrasound imaging of tumors before and after ultrasound stimulation following intratumoral injection of GB@vPCs (O_2_); b) Photoacoustic imaging of tumors with PBS injection acquired before and after ultrasound stimulation.

**Figure S16.** Body weight changes over time in mice following different treatments.

**Figure S17.** H&E staining of heart, liver, spleen, and kidney tissues on day 20 of treatment.

**Figure S18.** Representative gating strategy for identifying CD4⁺ and CD8⁺ T‑lymphocytes in the tumor tissues.

**Figure S19.** Representative gating strategy for identifying mature dendritic cells from lymph nodes.

**Figure S20.** Representative gating strategy for Naïve, central‑memory (TCM) and effector‑memory (TEM) CD8⁺ T‑cell subsets isolated from the spleen.
